# What Factors Affect Voluntary Uptake of Community-Based Health Insurance Schemes in Low- and Middle-Income Countries? A Systematic Review and Meta-Analysis

**DOI:** 10.1371/journal.pone.0160479

**Published:** 2016-08-31

**Authors:** David Mark Dror, S. A. Shahed Hossain, Atanu Majumdar, Tracey Lynn Pérez Koehlmoos, Denny John, Pradeep Kumar Panda

**Affiliations:** 1 Micro Insurance Academy, New Delhi, India; 2 Erasmus University Rotterdam, Rotterdam, Netherlands; 3 International Centre for Diarrheal Disease Research, Dhaka, Bangladesh; 4 The Uniformed Services University of the Health Sciences, Bethesda, MD, United States of America; 5 Peoples Open Access Education Initiative (Peoples-Uni), Delhi, India; 6 International Institute of Health Management Research, Dwarka, Delhi, India; University of British Columbia, CANADA

## Abstract

**Introduction:**

This research article reports on factors influencing initial voluntary uptake of community-based health insurance (CBHI) schemes in low- and middle-income countries (LMIC), and renewal decisions.

**Methods:**

Following PRISMA protocol, we conducted a comprehensive search of academic and gray literature, including academic databases in social science, economics and medical sciences (e.g., Econlit, Global health, Medline, Proquest) and other electronic resources (e.g., Eldis and Google scholar). Search strategies were developed using the thesaurus or index terms (e.g., MeSH) specific to the databases, combined with free text terms related to CBHI or health insurance. Searches were conducted from May 2013 to November 2013 in English, French, German, and Spanish. From the initial search yield of 15,770 hits, 54 relevant studies were retained for analysis of factors influencing enrolment and renewal decisions. The quantitative synthesis (informed by meta-analysis) and the qualitative analysis (informed by thematic synthesis) were compared to gain insight for an overall synthesis of findings/statements.

**Results:**

Meta-analysis suggests that enrolments in CBHI were positively associated with household income, education and age of the household head (HHH), household size, female-headed household, married HHH and chronic illness episodes in the household. The thematic synthesis suggests the following factors as enablers for enrolment: (a) knowledge and understanding of insurance and CBHI, (b) quality of healthcare, (c) trust in scheme management. Factors found to be barriers to enrolment include: (a) inappropriate benefits package, (b) cultural beliefs, (c) affordability, (d) distance to healthcare facility, (e) lack of adequate legal and policy frameworks to support CBHI, and (f) stringent rules of some CBHI schemes. HHH education, household size and trust in the scheme management were positively associated with member renewal decisions. Other motivators were: (a) knowledge and understanding of insurance and CBHI, (b) healthcare quality, (c) trust in scheme management, and (d) receipt of an insurance payout the previous year. The barriers to renewal decisions were: (a) stringent rules of some CBHI schemes, (b) inadequate legal and policy frameworks to support CBHI and (c) inappropriate benefits package.

**Conclusion and Policy Implications:**

The demand-side factors positively affecting enrolment in CBHI include education, age, female household heads, and the socioeconomic status of households. Moreover, when individuals understand how their CBHI functions they are more likely to enroll and when people have a positive claims experience, they are more likely to renew. A higher prevalence of chronic conditions or the perception that healthcare is of good quality and nearby act as factors enhancing enrolment. The perception that services are distant or deficient leads to lower enrolments. The second insight is that trust in the scheme enables enrolment. Thirdly, clarity about the legal or policy framework acts as a factor influencing enrolments. This is significant, as it points to hitherto unpublished evidence that governments can effectively broaden their outreach to grassroots groups that are excluded from social protection by formulating supportive regulatory and policy provisions even if they cannot fund such schemes in full, by leveraging people’s willingness to exercise voluntary and contributory enrolment in a community-based health insurance.

## Introduction

Healthcare-seekers, through out-of-pocket (OOP) spending at the time and place of treatment, defray most healthcare costs in developing countries. In India, private funds pay for 70 percent of all healthcare, and OOP spending represents 86 percent of it [1,2]. This inequitable and inefficient health financing situation pervades other LMICs as well. The solution proposed by the World Health Organization (WHO) and other international bodies has been to strive toward universal health coverage (UHC), notably through health insurance. However, few low-income countries have been able to mount mandated funding solutions for UHC that truly cover their entire populations [3]. Attempts to subsidize large population segments have been rare and stopped short of UHC [4,5].

One way to enhance coverage could be through Community Based Health Insurance (CBHI) schemes [6], which are local mutual aid schemes that put in place arrangements for mobilizing, pooling, allocating and managing or supervising members’ resources for healthcare [7, 8]. CBHI schemes have been effective in reducing OOP and improving access to healthcare services [9]. However, such schemes are usually small. If CBHI should be viewed as part of social protection policies, such schemes will have to move from niche to scale by enlisting and retaining more members.

To see how this might happen, the *International Initiative on Impact Evaluation* (3ie) commissioned a Systematic Review of the literature, and the *Evidence for Policy and Practice Information and Co-ordinating Centre* (EPPI-Centre) assisted the authors in elaborating the Protocol [10]. This article, summarizing the full report [11] answers the following questions relating to voluntary uptake (and renewal) of CBHI enrolment in LMICs

1. Demand-side factors
  - Which household and individual characteristics affect CBHI uptake?
  - Which social-capital–related factors in the community affect uptake?
2. Supply-side factors
  - Which scheme-related factors affect access to CBHI?
  - Which institutional factors (e.g., governance, marketing, self-help group (SHG) membership) play a role in increasing uptake?
  - Which other health-related supply-side factors enhance CBHI uptake?
3. What factors affect CBHI renewal and retention of clients?

Previous systematic reviews of the literature on CBHI bear testimony to the persistent interest in this health financing mechanism over many years. Ekman [12] examined 15 peer-reviewed articles plus 21 unpublished papers and reports (grey literature) dating from 1980 to 2003, in English, French, Portuguese and Spanish. Ekman concluded that there was then strong evidence that CBHI provided some financial protection by reducing OOP spending, moderate evidence that such schemes improved cost-recovery and that there was still insufficient agreement on outcome indicators that should be followed. Robyn PJ, Sauerborn R, Bärnighausen T. [13] conducted a systematic review of 34 publications (dealing with 17 schemes in South Asia, 10 in sub-Saharan Africa, 4 in East Asia and 1 in Latin America) on provider payment methods used by CBHI, published up to January 2010. These authors concluded that the evidence on the influence of one of five provider payment systems (fee-for-service, salaries, coverage ceiling, capitation and co-insurance) on customer demand and population enrollment, risk pooling or financial sustainability of CBHI was limited and not particularly strong, as most studies were observational rather than based on trials or on quasi-experimental research. The most recent systematic review, by Adebayo et al [14] included 25 studies published in English between 2003 and 2013 (18 quantitative, six qualitative and one mixed method) on factors affecting uptake of CBHI in LMIC, many of which were about measuring willingness to pay. Considering that studies dealing with WTP must be treated as unreliable sources of evidence of actual enrolment, we applied a different selection criterion whereby WTP studies were included in our review only when they also dealt with willingness-to-enrol, as our investigation is about factors affecting uptake (enrolment) and renewal of membership in CBHI. Thus, Readers asking *why do we need another review for CBHI*? will be interested to know that 44 out of the 54 included in our review were considered only in our review, and only 10 articles were considered for the review also by Adebayo et al. Moreover, our study is the first and only following the rigorous research protocol that has been approved by EPPI centre and 3ie that included both thematic synthesis of qualitative articles and meta-analysis of quantitative studies covering many more publications (reflecting notably a longer time-frame and four languages rather than only one), thus obtaining stronger external validity of the findings and conclusions on this topic. We also show, in the conclusion section of this article, how our conclusions differ from those of earlier systematic reviews of the CBHI literature.

## Methods

### Information Sources

The literature search (conducted from May to November 2013) included academic and gray literature. The academic thematic databases included social science, economics and medical sciences (Business Source Premier [EBSCO], Econlit [EBSCO], Global health [OVID], ISI web of knowledge, Medline [OVID], ProQuest, Scopus, Sociological Abstracts) and other electronic resources (e.g., Eldis and Google Scholar). The gray literature was searched manually, through citation tracking and personal communications.

### Search Strategy

Following the PRISMA protocol (S1 Table), search strategies for electronic databases were developed by one of the authors (SH) and peer-reviewed by search specialists at 3ie and EPPIA. The complete Medline/PubMed search strategy is appended (S1 Text). The search identified studies published from 1990 to 2013, in English, Spanish, French and German. The search strategy was translated by an information specialist for use in other databases using the appropriate controlled vocabulary. Electronic search results or publications available digitally in ‘.ris’ format were uploaded to review software (*EPPI-Reviewer 4*), for screening, reviewing, coding and further processing by the review team.

### Eligibility Criteria (Inclusion/Exclusion)

The *inclusion criteria* were determined by “*PICOS*”. **P**articipants invited to join CBHIs voluntarily in LMICs (World Bank definitions 2012) were included, whether they decided to enroll or not. Also included were voluntary, contributory and community-based **I**nterventions (when in LMICs); **C**omparisons between individuals who do and who do not join CBHI schemes and those who renew or dropout); **O**utcomes when related to determinants of demand (e.g., socioeconomic characteristics or social capital in the community) or supply (e.g., scheme-related factors, institutional factors or other health-related factors that enhance CBHI uptake). The review also included factors affecting renewal (or dropout) in CBHI schemes. **S**tudy design influenced the inclusion. Among quantitative studies included were randomized controlled trials, quasi-experimental studies, experimental designs with control groups, and observational studies (quantitative surveys, cohort studies, case-controlled studies and case studies) that dealt with factors affecting enrolment and renewal/dropout. The qualitative studies included case studies, interviews/key informant interviews (KIIs), and focus groups with participants (who enrolled, did not enroll, renewed or dropped out) and scheme managers/policymakers.

Studies were *excluded* if (a) published before 1990; (b) a policy analysis or opinion piece; (c) dealing with a non-LMIC country; (d) dealing with other health insurance mechanisms (private, social or mandatory); and (e) examining only the impact of already functioning CBHI schemes.

### Data Extraction

Using a coding tool characterizing studies by context, mechanism and outcomes (S2 Text: Data Extraction Sheet), two researchers independently extracted information about study characteristics (e.g., research country, focal research area, population characteristics, study design, sample size, analytical framework and findings).

### Critical appraisal of studies: quality assurance process

Quality assurance was maintained in six stages: (1) potential citations were imported to *EPPI-Reviewer 4* and duplicates removed; (2) studies were scanned on the basis of title and abstract, (3) and (4) two of the authors independently appraised the studies; any discrepancies in the critical appraisal were resolved through discussion, and any issue that could not be resolved was discussed with a third author (5) studies retained were consolidated with studies published after the search strategy had been applied, and these studies were added manually; (6) the assessment of the “Remaining Included Studies” was done following checklists for critical appraisal of quality (S2 Table) and separated into:

1. *Randomized control trial studies*: Risk of Bias Assessment Tool, Table 8.5d –Cochrane Handbook for SRs of Interventions [15]; *Cohort studies*: Critical Appraisal Skills Programme (CASP) checklist for cohort studies [16]; *Quantitative studies* (case-control and cross-sectional): Critical Appraisal Checklist [17].
2. *Qualitative studies*: Critical Appraisal Skills Programme (CASP) checklist for qualitative studies [18,19]
3. *Mixed method studies*: Quantitative and qualitative components of the study were judged on quality using their respective checklist adopted for each component separately.

### Analysis: Overall Approach to Synthesis

The studies retained for detailed analysis were processed through four stages: (1) *coding of quantitative studies* (including quantitative data from mixed-method studies) and qualitative studies (including qualitative data from mixed-method studies) by key features (study objectives, design, sample size, analytical methods, context, and findings) (S3 Table). (2) *a meta-analytic synthesis* of the quantitative studies (hereafter meta-analysis), with subtopics as follows:

*(2A) Estimating effect size*: Most studies included in this meta-analysis reported odds ratios or the coefficients of regression of the logit or probit model [20]. We used the following formulas to convert these measures into the effect size.

$$Effect\;Size=\;\frac{\text{ln}\left(OR\right)}{1.81}\;,\;where\;\;OR\;\;is\;\;the\;\;odds\;\;ratio\;\;estimated\;\;by\;\;a\;\;logit\;\;model$$

$$Effect\;\;Size=\;\frac{Coefficient\;\;of\;Regression}{1.81}\;,\;where\;\;the\;\;coefficient\;\;of\;regression\;\;is\;\;estimated\;\;by\;\;a\;\;logit\;\;model$$

Effect  Size= Coefficient  of Regression , where  the  coefficient  of  regression  is  estimated  by  a  probit model

*(2B) Estimating standard error of the effect size*: Standard error (SE) of the effect size was estimated from the SE of the odds ratio or the coefficients of regression of Logit or Probit model, by applying similar transformation used for estimating the effect size. Some authors reported the 95 percent confidence intervals instead of the SE. For these studies we first computed the 95 percent confidence interval of the effect size by applying similar transformations and then computed the SE of the effect size by using the following formula.

$$Standard\;Error\;of\;the\;Effect\;Size=\;\frac{95\;percent\;\;CI\;of\;ES\;\left(Upper\;Limit\right)-95percent\;CI\;of\;ES\;\left(Lower\;Limit\right)}{2*\;1.96}$$

Some studies did not report the confidence intervals but reported the t-statistic for the coefficient of regression. For these studies we first estimated the SE of the coefficient of regression by using the following formula

$$Standard\;Error\;of\;the\;Coefficient\;of\;Regression=\;\frac{Coefficient\;of\;Regression}{t\;statistic}$$

and then estimated the SE of the effect size by using the same transformation used to estimate effect size.

Some authors did not report the SE, CI (Class Interval) or the t-statistic. Therefore, we could not estimate the SE of the effect size for these studies [21,22].

*Sample size*: Sample size was reported by all the studies.

*Weights*: The standard practice in meta-analysis is to apply weights *proportional to the variances* of the effect size for estimating the summary effect. But this could not be applied in this meta-analysis exercise as the SEs of the effect size for a few studies could not be estimated. It was also not wise to exclude because they were based on large samples. Therefore, we applied weights *proportional to the sample size* to estimate the summary effect by combining the effect size estimated from individual studies.

*(2C) Estimating summary effect*: When a characteristic or a trait influencing a household’s enrolment behavior was reported in the same way by all studies, the summary effect was obtained by averaging the effect sizes, after applying weights. However, the studies included in this meta-analysis reported the same characteristic in different ways (e.g., as continuous and categorical variables), and authors used heterogeneous categories for analyzing data when it was a categorical variable (Box 1).

**Box 1. Heterogeneity across Studies: The Major Challenge to Meta-Analysis**

Oriakhi (Edo State, Nigeria), Kuwawenaruwa (Tanzania) and Panda (India, 3 sites) used the household head’s age as a continuous variable in logit model. Other authors used that age as a categorical variable. But different authors had different base categories and estimated odds ratios for multiple but non-uniform categories. Ranson (Gujrat, India) assumed three categories of age (18–29 years (base); 30–39 years; and 40 years and above). Gumber (Gujrat, India) assumed five categories for age: (16–25 years (base); 26–35 years; 36–45 years; 46–55 years; and 56 years and above). De Allegri (Burkina Faso, 2006) created three categories (20–40 years (base); 41–60 years; and 61 years and above). Gnawali (Burkina Faso, 2009) also used three categories (the same base category as Allegri; 41–64 years; and 65 years and above). Schneider (Rwanda) had two (Below 40 years (base) and 40 years and above). Chankova (Ghana, Mali and Senegal) created four categories (less than 40 years (base); 40–49 years; 50–59 years; and 60 years and above). Mathiyazaghan (Karnataka, India) mentioned three categories: youthful (base), middle-aged and old-aged, without any mention of age-brackets. Based on a thorough literature search we concluded that in the Indian context youthful was 15 to 29 years; middle aged = 30 to 59 years; and old aged were 60 years and above.

Handling this heterogeneity was the major challenge of the meta-analysis. Averaging the effects across very heterogeneous studies with weights was not an option. Instead, we applied an innovative technique to obtain the summary effect. Effect size for a continuous variable is basically the transformation of the regression slope line, and it implies the amount of increase in the effect size for unit increment in the independent variable. Thus, the effect size for a particular category of a categorical variable should be uniform for that category. Multiple categories can be considered as a step function of the independent variable. To combine the effect sizes estimated from individual studies, we first simulated the effect sizes from each study over a domain of interest (= range of values of the independent variable), then merged them in a single dataset and fitted a linear regression over the merged dataset. We consider the coefficient of regression (of the merged dataset) as the summary effect, the average increase in the effect size for unit increment in the independent variable, now a combination of continuous and categorical variable. We applied this technique to estimate the summary effect of four variables: household head’s age and educational status, household size and income/expenditure/assets quintile. This method has been referred to as the regression method for obtaining summary effect.

Standard Error (SE) of the summary effect estimated using the regression method has been computed by Stata with the following command:

Regress {Dependent Variable} {Independent Variable} [Pweight = SampleSize]

(3) In the third stage, we performed a thematic synthesis of qualitative studies (hereafter thematic synthesis) following [3]. First, two researchers’ independently reviewed and analyzed quotations and other relevant texts and developed codes by labeling the data. Second, codes were redefined with additional data. These codes led to the thematic framework for examination of common elements across studies. Finally, theme charts were populated by all the studies, an analytical framework was developed and findings were analyzed to explain factors associated with enrolment or renewal (dropout) decisions.

(4) In the final stage, the qualitative synthesis (informed by thematic synthesis) and quantitative synthesis (informed by meta-analytic synthesis) were compared, to gain insight for an overall synthesis of findings/statements.

## Results

### Study Selection

**Fig 1:**
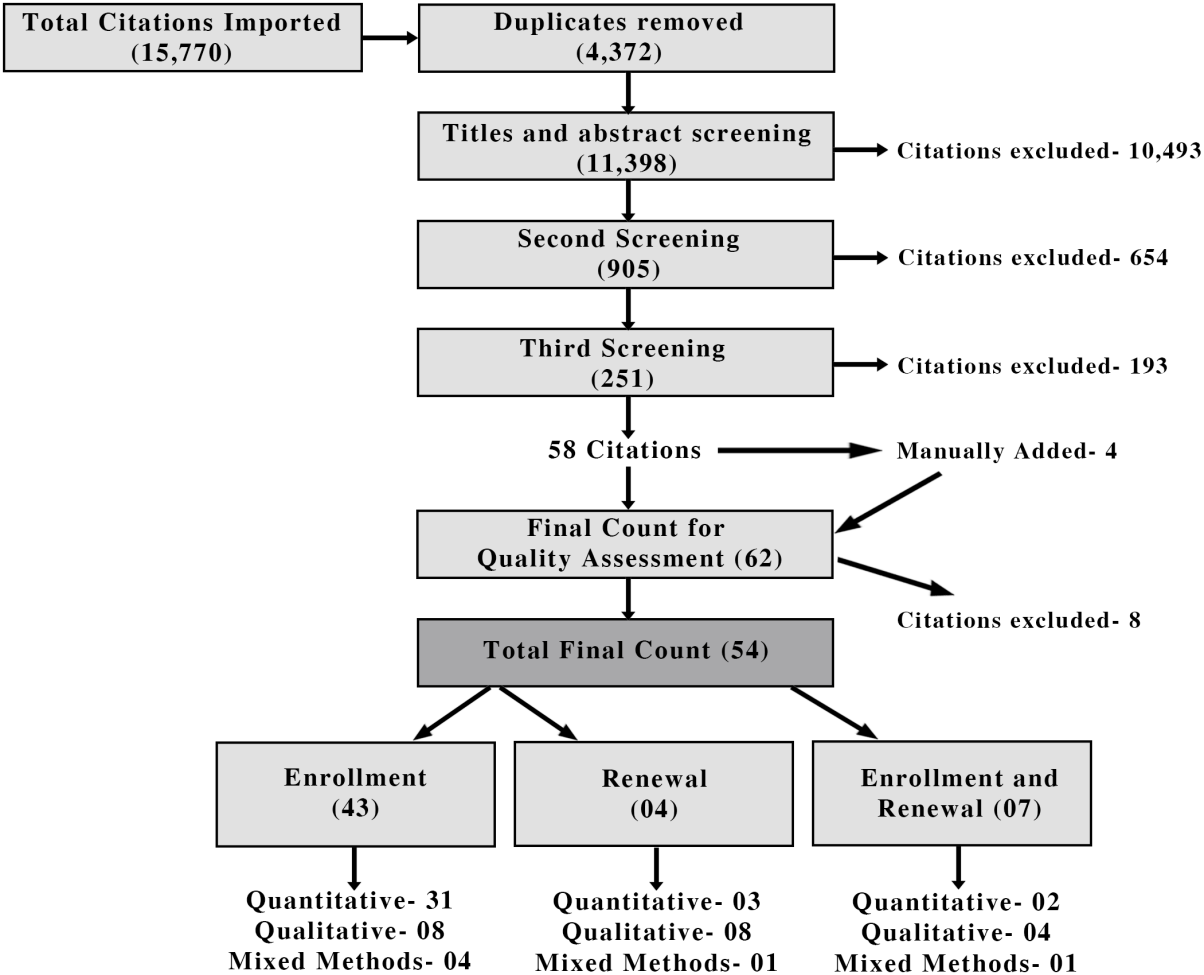
PRISMA Flowchart Diagram of Study Selection.

The flow of study selection is shown in Fig 1.

### Study Characteristics

The 54 papers [21–74] describing 56 countries retained for the SR (listed in S3a, S3b and S3c Table) present evidence from 20 countries (Afghanistan, Armenia, Benin, Burkina-Faso [x7], Cambodia, Cameroon [x2], China [x3], Democratic Republic of Congo, Ecuador, Ghana [x5], Guinea-Conakry, India [x9], Malaysia, Mali, Nigeria [x7], Rwanda [x2], Senegal [x4], Sri Lanka, Tanzania [x3], and Uganda [x4]). The largest number of CBHI uptake studies was conducted in Sub-Saharan Africa (SSA), followed by Asia, with only few studies in other countries. Thirty-two studies were conducted in rural settings, 7 in an exclusively urban environment, and 15 involved both rural and urban settings. Most studies were conducted in low-income countries, a few studies in lower-middle income countries, and 3 studies in upper-middle income countries (China, Malaysia and Ecuador). Of the 54 studies, 4 examined CBHI schemes operating at national level, 39 at regional level and 11 at local level. All 7 studies in Burkina-Faso dealt with the Nouna Health District CBHI scheme (regional level). Studies in Nigeria studied mostly the schemes in Anambra and Enugu districts.

In terms of timing, CBHI research has taken off only in the last decade: few studies were undertaken in the 1990s, and most publications date to 2005–06 and 2009–11.

Out of the 42 quantitative studies (36 quantitative [21,23,27–29,36–37,39–47,49–52,54–59,61,63,65–66,68–69,73–74] and 6 mixed methods with quantitative (and qualitative) data [25,38,60,62,70,72]) one dealt with a randomized control trial (RCT), 5 with case-control methods and cohort studies, and the remaining 31 were cross-sectional surveys based on random samples. Twenty-nine studies examined correlates of uptake and renewal using multivariate analyses (logit/probit/tobit). One study was based on panel data, and used fixed effect and random effect models. Eleven studies used descriptive statistics with statistical tests, and one descriptive study used no statistical test.

Out of the 18 qualitative studies (12 qualitative [30–35,48,64,67,71,75–76] and 6 mixed-methods with qualitative (and qualitative) data [25,38,60,62,70,72]), 10 used both focus groups and interviews, and four each used either focus groups or interviews with different participants (insured, uninsured, renewed or dropped out) and with scheme managers and healthcare providers to elicit in-depth understanding of the reasons for uptake and renewal in CBHI schemes.

### Results of Quantitative Synthesis (Meta-Analysis)

Meta-analysis was conducted for 18 [21–23,28,35,40,44–45,49–50,52,61,63,65,68–69,73–74] quantitative studies. We studied in depth the following variables:

- Socioeconomic status of household
- Presence of acute illness in household
- Presence of chronic illness in the household
- Household head’s educational level
- Household size
- Household head’s marital status
- Household head’s age
- Presence of elderly people (above 65 years) in household
- Household head’s gender
- Trust in insurer

The results of the meta-analysis (pooled effect size and 95 percent confidence intervals) are summarized in Table 1 for enrolment and Table 2 for renewal/dropout. We report the summary effects for each region separately and also after clubbing them together, for enrolment and renewal/dropout decisions.

**Table 1:** Summary Effects for the Variables Influencing Enrolment (Combined Regional and Overall).

Variables	Summary effect			Method of estimating summary effect
	Asia	Sub-Saharan Africa	All	
Socioeconomic percentile	0.2379	0.5209	0.4626	Regression
Presence of acute illnesses	0.1169	-	-	Averaging
Presence of chronic illnesses	0.0909	0.0495	0.0597	Averaging
Household head’s educational level	0.0153	0.0555	0.0443	Regression
Household size	-0.0036	0.0414	0.0323	Regression
Household head’s marital status	0.1543	-0.0027	0.1403	Averaging
Household head’s age	0.0082	0.0042	0.0048	Regression
Presence of elderly person	-0.1847	-0.1614	-0.1731	Averaging
Gender	-0.0635	-0.4083	-0.3556	Averaging

**Table 2:** Summary Effects for the Variables Influencing Renewal/Drop-Out.

Variable	Type of variable	Summary effect		
		Asia	Sub-Saharan Africa	All location combined
Household head’s gender	Cat: Female = 0, Male = 1	0.4500	0.0072	0.1581
Trust in insurer	Cat: No trust = 0, trust = 1	0.1800	0.7700	0.5076
Household size	Con	0.0200	-0.0400	0.0135
Household head’s education	Con: years of education	0.0542	0.013	0.0460
Socioeconomic status	Con: socioeconomic percentile	-	-0.0341	-

The results show that the determinant influencing enrolment most in CBHI, in both regions, is socioeconomic status (SES) of households. Next is heavy incidence of chronic illnesses in households, which are more likely to join the CBHI (the effect is stronger in SSA than in Asia). Similarly, educated, mature and female household heads attach more value to CBHI membership; gender matters most, followed by education and age. Presence of elderly people negatively influences enrolment. The two variables that behave differently across the two regions are household size and Household head’s marital status. Household size has a negative effect in Asia but a positive effect in SSA. The effect of Household head’s marital status is close to zero (negative) in SSA and positive and somewhat higher in Asia. These results suggest that SES, education, gender and age have similar effects universally, whereas marital status and household size have only localized impact.

The direction of the impact on enrolment is the same for most variables (except household size and marital status).

As regards renewal/dropout decisions, trust in the insurer had the largest effect, followed by male heading of household and household head’s education. While trust in the insurer had a stronger effect in SSA, household head’s gender and education had a greater influence on renewal decisions in Asia.

The small R-square values for the fitted regressions (estimating the summary effects) for some of the variables indicate localized behaviors may rule out any meaningful combining of the results of several studies.

In the next section more details are presented on each variable considered in the meta-analysis.

#### Socioeconomic Status of Household

**Fig 2:**
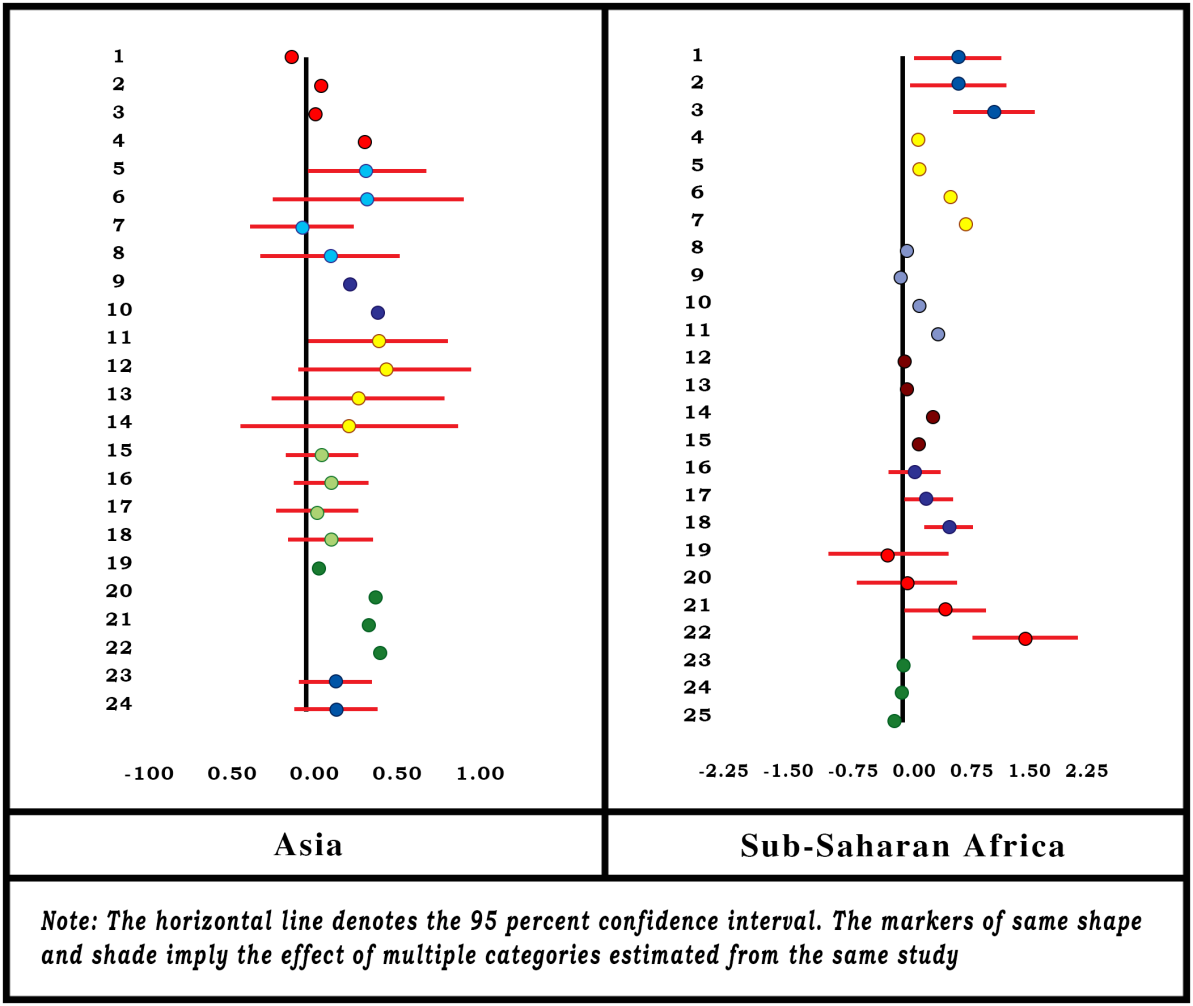
Forest-Plot for Socioeconomic Status.

Ten studies [21–22,35,44–45,50,63,65,68,73] reported that the key variable influencing enrolment in CBHI was the SES, but different authors use different variables as reference: Some refer to income as the indicator of SES; others consider expenditure level; and yet others construct socioeconomic categories based on household assets. We presume that notwithstanding the differences between income, expenditure and assets, all three can throw light on the SES of a household, and we consider the categories based on either of them (in absence of any uniform measure) as an indicator of SES of a household. See Fig 2.

The forest-plot diagram for the variable indicates a positive association between household SES and the likelihood of its joining the CBHI. It can also be seen that the effect size increases as SES increases in SSA, but the shift in the effect size is not unidirectional in Asian locations. Effect is positive when the household ranks above the poorest category, but that effect size diminishes as the household SES is higher.

Authors classified the SES categories of households based on income, expenditure or asset, but the categories were not identical across all studies. For instance, some classified the households on the basis of tertiles, others on the basis of quartiles, and some created quintiles. The heterogeneity of these categories made it impossible to use a standard methodology to estimate the summary effect. Hence we assumed (i) uniform effect size within a given category (tertile, quartile or quintile) and (ii) the distribution of households over the domain of socioeconomic *percentile* (instead of tertile, quartile or quintile). We then applied the regression method of obtaining summary effect by fitting a linear regression of effect size on the percentile values.

The SE of the summary effect for all locations combined is 0.001 which implies that the effect is significantly different from zero (p-value = 0.000). We estimate the summary effect of socioeconomic percentile for Asian and SSA locations as 0.258 and 0.5209, respectively (Table 1).

In line with the meta-analysis, similar results appear in findings of all the quantitative studies. In 84 percent of quantitative studies, HH socioeconomic status is positively associated with enrolment.

#### Presence of Acute Illnesses in Household

**Fig 3:**
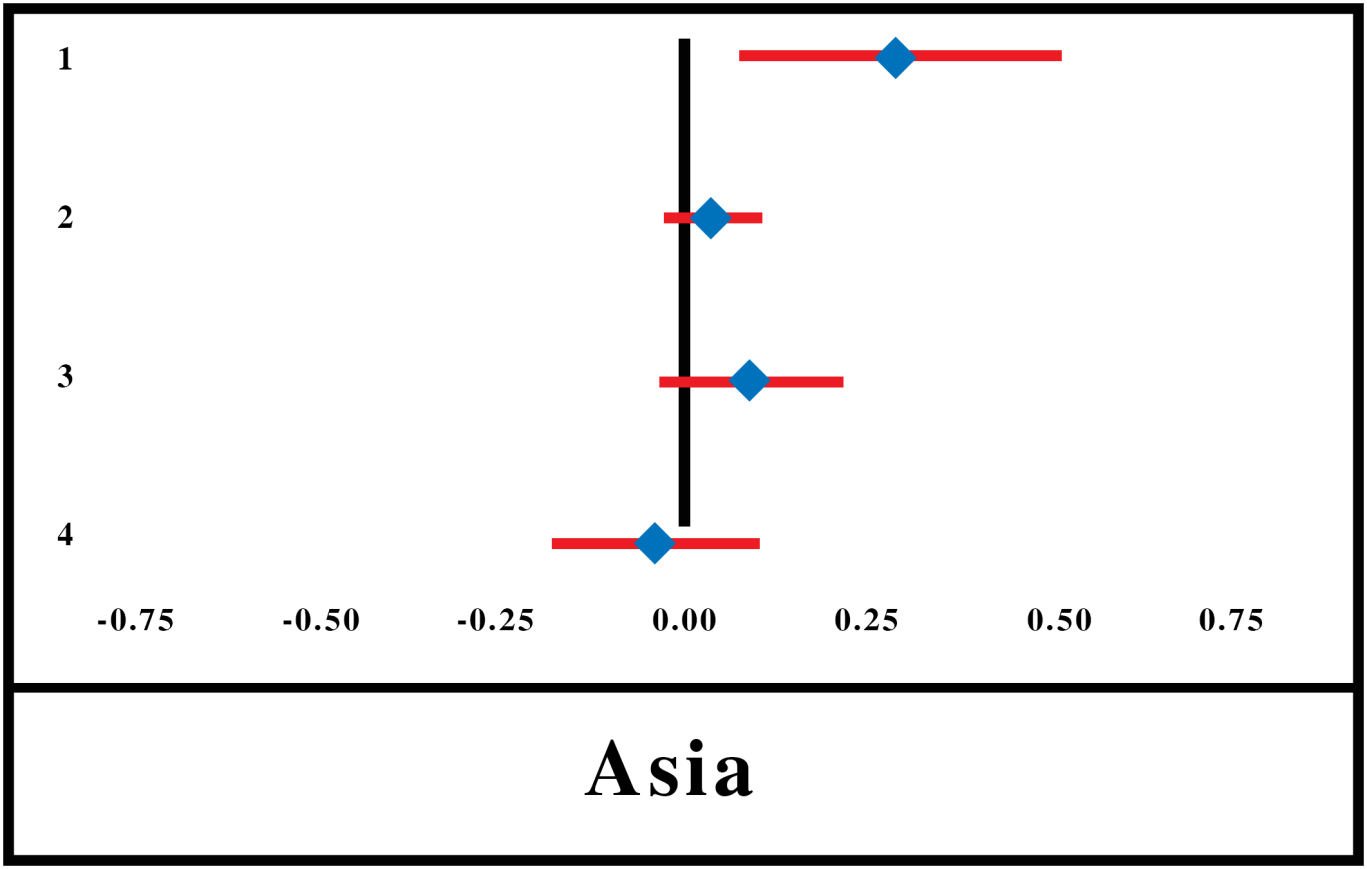
Forest-Plot for Presence of Acute Illnesses in Household.

Only 2 studies [63,65] involving 4 Asian locations (and none from SSA) reported on acute illnesses (during the month preceding the survey) as a determining factor for enrolment in CBHI. One of those showed a positive association between the presence of acute illnesses and enrolment, and this effect was significantly higher than zero. Acute illnesses in the household were treated as a continuous variable, estimated at 0.138. However, the results are only indicative and not conclusive as the SE for the summary effect size could not be calculated (Fig 3). In four-fifths of the full range of quantitative studies, enrolment is positively associated with the presence of acute illness in the household, suggesting similar results from meta-analysis and vote count.

#### Presence of Chronic Illnesses in Household

**Fig 4:**
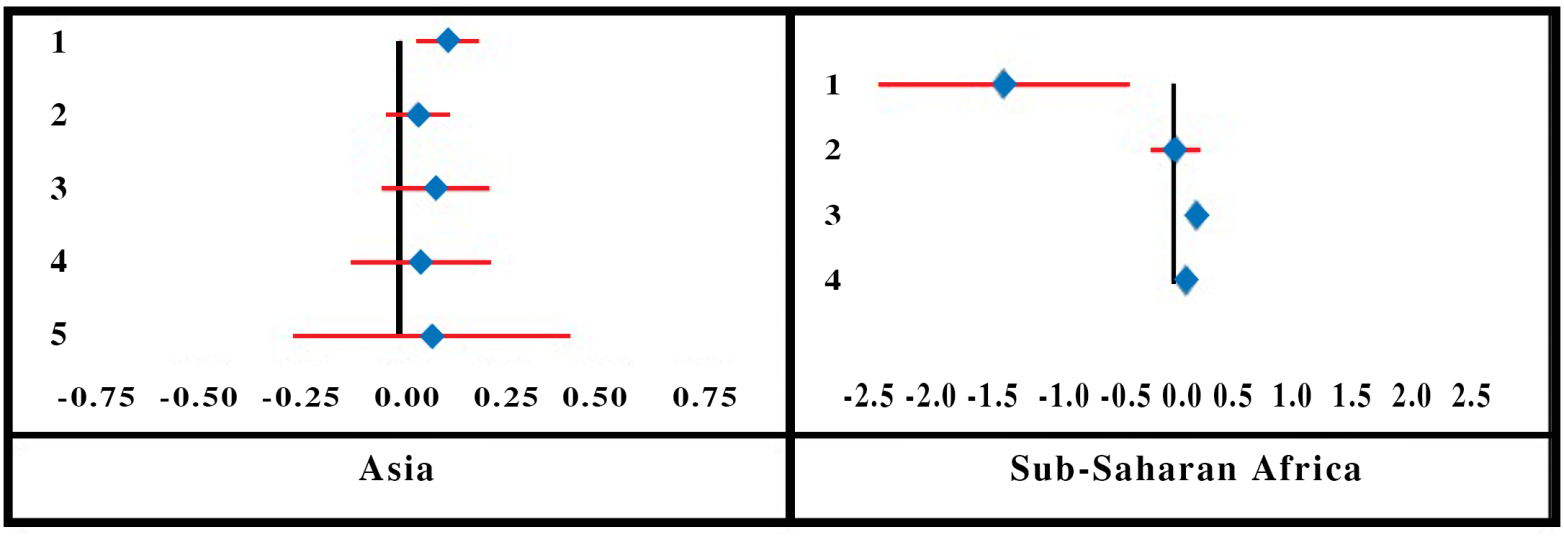
Forest-Plot for Presence of Chronic Illnesses in Household.

Three studies [23,45,63] involving 5 Asian locations and 3 studies [21,35,44] involving 4 locations in SSA reported the effect of chronic illnesses in the household on enrolment. The forest-plot is displayed in Fig 4. The Asian studies report a positive association between chronic illnesses in the household and enrolment in CBHI, but only one was significantly different from zero. In SSA the effects are close to zero and one is significantly negative. We estimate the summary effects 0.097, 0.0495 and 0.0601, respectively, for Asia, SSA and all locations combined together (Table 1).

#### Household Head’s Education

**Fig 5:**
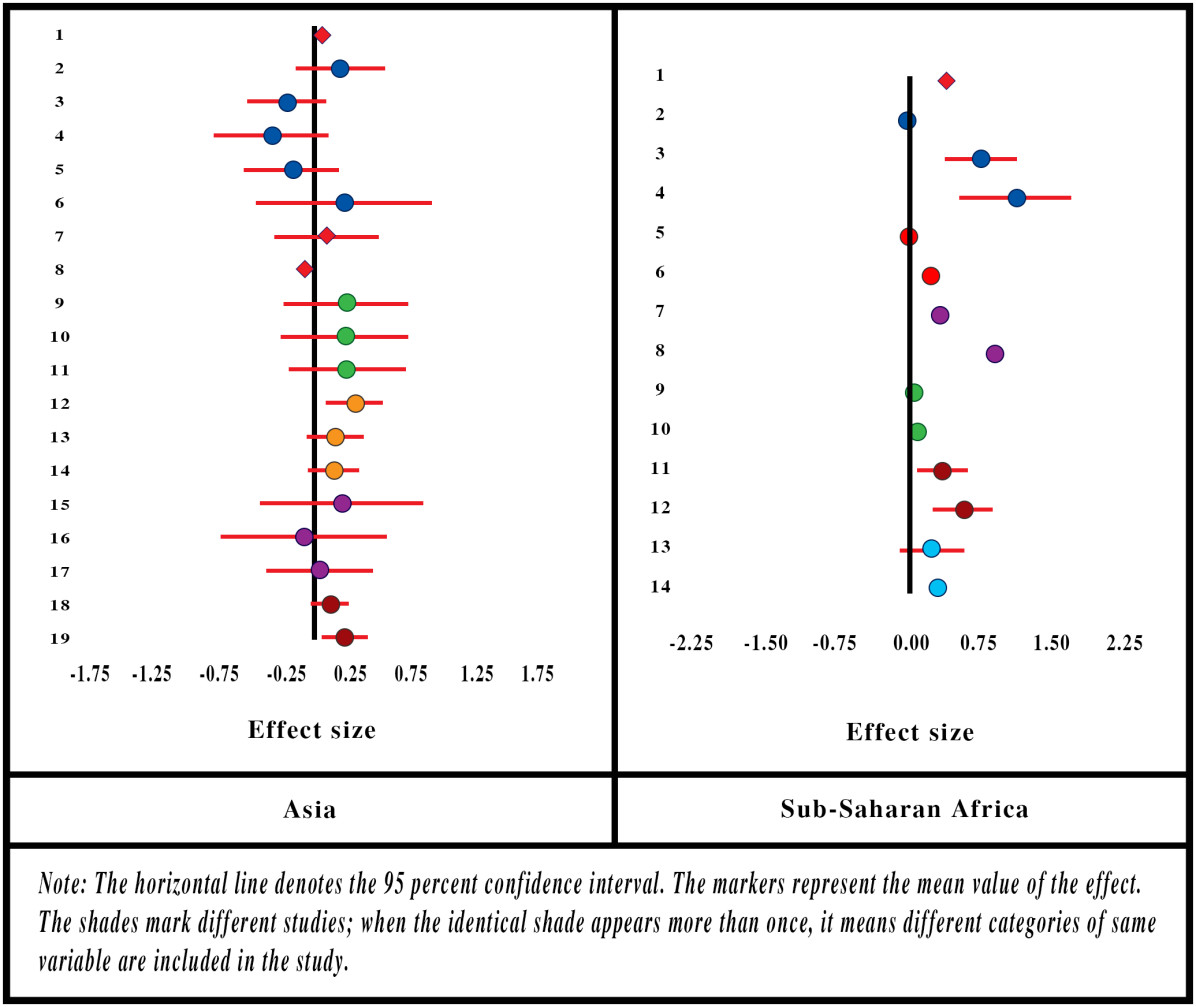
Forest-Plot for Household Head’s Education.

Many studies reported a positive association between household head’s educational level and enrolment in CBHI, and the effect size is apparent in the forest-plot diagram (Fig 5).

Some authors did not report the SE of their results, and others reported seemingly counter-intuitive results, whereby with increased educational level, the effect size is reported to be both positive and negative. However, as a whole the association appears positive, with effect size not always significantly different from zero.

Three authors [23,61,68] measured education in terms of number of years in school, and considered it a continuous variable. The rest [10,21, 22,35,44,45,49,50,65,73] treated it as a categorical variable. All of them assumed the same base category (no experience of schooling), but dealt with multiple heterogeneous categories for the educational level (e.g., primary, secondary, middle, upper-middle). To estimate the summary effect of Household head’s educational level, we applied the regression method for obtaining summary effect (as described in the methods section) over a domain of 0 to 15 years of schooling. The summary effect size of education, after applying weights (proportional to the sample sizes), is estimated as 0.0167 for Asia, 0.0555 for SSA and 0.0451 when two regions are clubbed together (Table 1). R-square for the weighted OLS is estimated at 0.069 (for two regions combined). SE of the summary effect for all locations combined is estimated at 0.0002, which implies that it is significantly different from zero (p-value = 0.000).

In line with meta-analysis, the vote count results of the full range of quantitative studies support the positive association between education of the head of household and enrolment in 81 percent of cases.

#### Household Size

**Fig 6:**
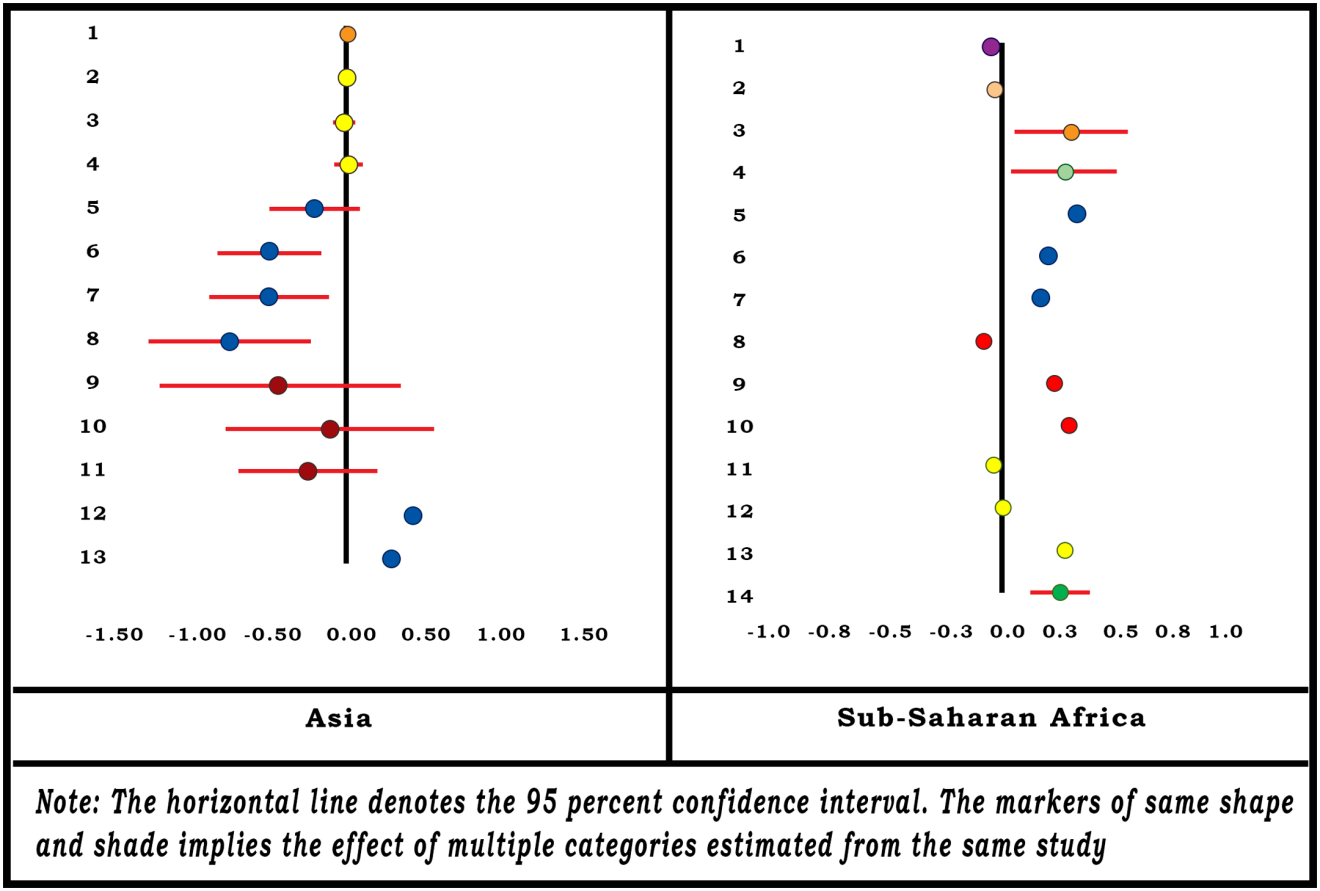
Forest-Plot for Household Size.

As is evident from the forest-plot diagram (Fig 6), that household size has a negative association with enrolment in Asia and a positive association in SSA. The variable has been treated differently by different authors—continuous as well as categorical with many non-uniform categories. Hence we apply regression method to estimate the summary effect—for regions as well as all locations combined and report the coefficient of regression as the summary effect.

We estimate the summary effects -0.0040 for the Asian locations [22,23,45,63,65] and 0.0414 for the SSA locations [21, 49,50,61,68]. The summary effect for all locations combined is estimated at 0.0328 with R-square value = 0.059 (Table 1) and SE = 0.0002. The small SE implies that the effect significantly differs from zero (p-value = 0.000).

The vote count findings of all quantitative studies suggest a positive relationship between household size and enrolment in three-fifths of the studies. However, the estimate of the summary effect in meta-analysis might have been influenced by large sample size used in some studies.

#### Household Head’s Marital Status

**Fig 7:**
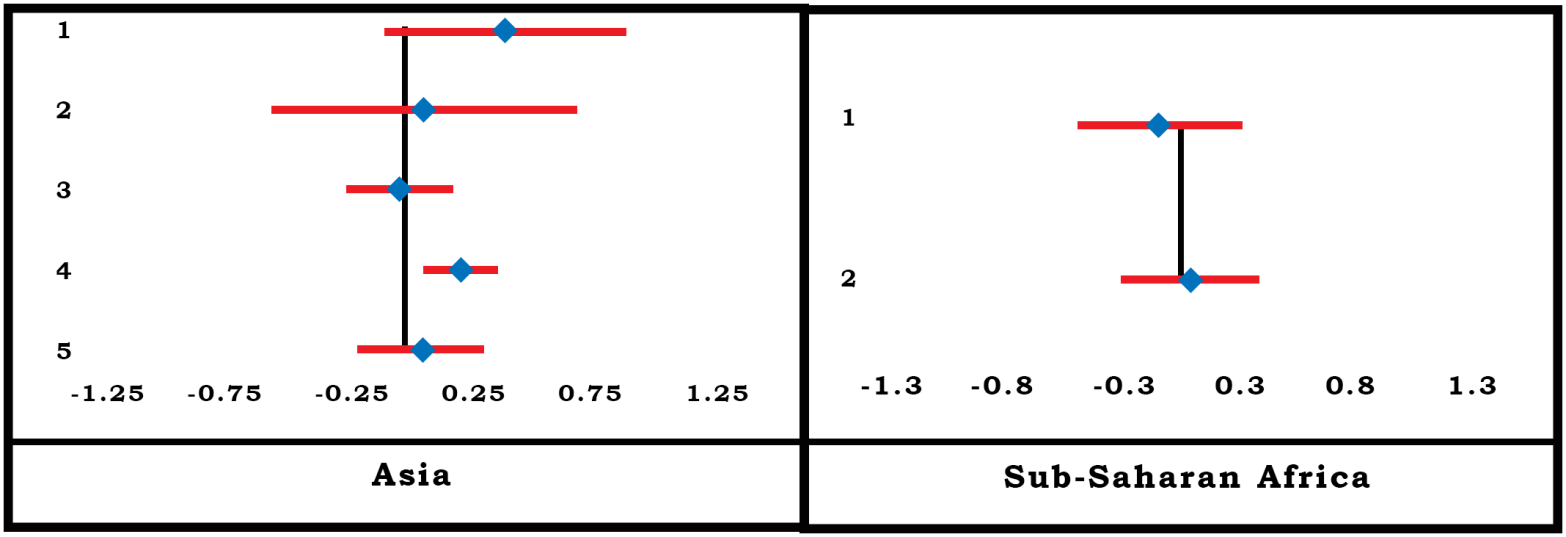
Forest-Plot for Household Head’s Marital Status.

Three studies [45,65,73] involving five locations from Asia and two studies [49,61] from SSA probed household head’s marital status as a determinant of enrolling in CBHI (Fig 7). Four studies from Asia and one study from SSA reported a positive association (a household with a married head is more likely to join the CBHI than one with an unmarried head). Overall summary effect for the variable is estimated positive for Asia (0.1543) and negative for SSA (-0.0027). Estimated summary effect for the variable for all locations combined was 0.1403 (Table 1). However, the results are only indicative and not conclusive as the SE for the summary effect size could not be calculated.

Similar to the results of the meta-analysis, vote count results suggest that 86 percent of all the quantitative studies found a positive association between households with a married household head and enrolment.

#### Household Head’s Age

Eight authors [21,22,35,44,45,63,65,68] have studied household head’s age associated with the enrolment in CBHI. Again, the variable was treated differently by different authors—continuous and categorical with heterogeneous categories.

**Fig 8:**
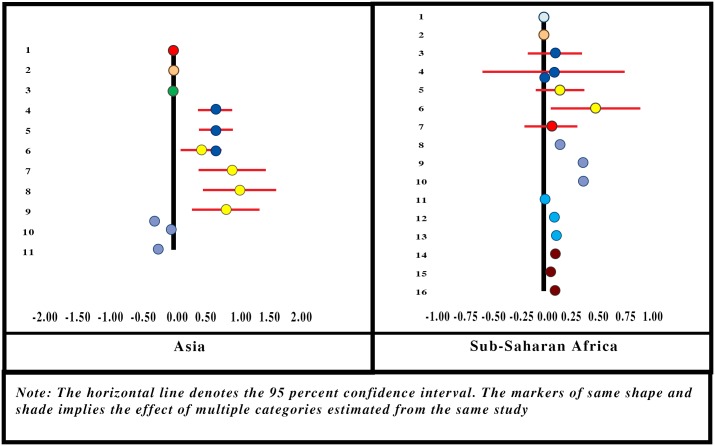
Forest-Plot for Household Head’s Age.

The forest-plot diagram for the household head’s age (Fig 8) indicates a positive association between the age of the head of household and enrolment in CBHI in SSA. In Asian locations, it is a mixture of positive and negative associations. For those studies where the age has been treated as a continuous variable, the slope of the regression is almost zero.

We apply the regression method to obtain summary effect and fit a regression of the effect size on household head’s age ranging from 16 to 65 years, with weights proportional to the sample size. We estimate the summary effect of the variable as 0.0048 with R-square value of 0.15 for all locations combined (Table 1). The SE of the summary effect is estimated to be very small (8.43E-06) implying that the effect significantly differs from zero (p-value = 0.000). Separate estimates of the summary effect for Asian and SSA locations are estimated at 0.0092 and 0.0042, respectively.

When the association between household head’s age and enrolment is considered in all quantitative studies, we find a positive relationship in half of the studies.

#### Presence of Elderly People in Household

**Fig 9:**
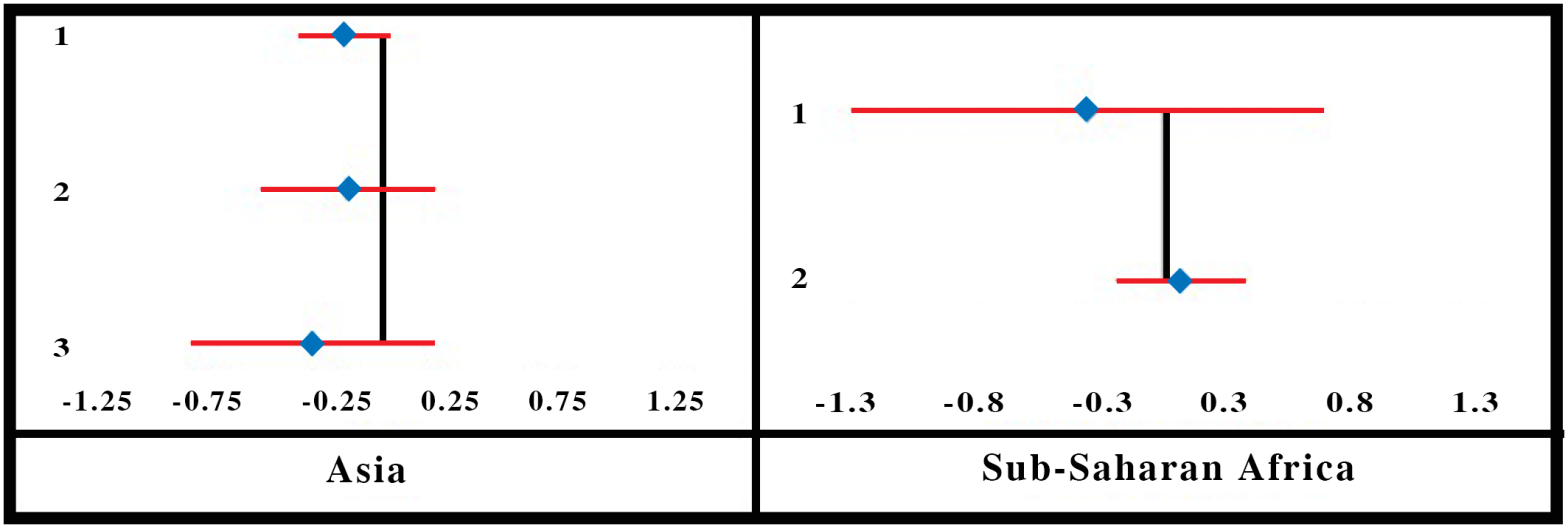
Forest-Plot for Presence of Elderly People in Household.

Only one study [63] involving three locations for Asia and two studies [44,49] from SSA probed the effect of elderly household members on enrolment in CBHI (Fig 9). All studies in the Asian locations indicate a negative association between the two. One of the two studies in SSA reported a negative association and the other reported a slightly positive association. Overall the summary effects are estimated to be negative for both regions (-0.212 for Asia and -0.1614 for SSA) and also for all locations combined (-0.181) (Table 1). However, the results are only indicative and not conclusive because the SE for the summary effect size could not be calculated.

The vote count results from the full range of quantitative studies contradict the findings of the meta-analysis. In three-fifths of all the quantitative studies, we find a positive association between enrolment and the presence of elderly people in the household while the relationship was negative in meta-analysis. This discrepancy could be explained by the small number of studies involved or that the vote count does not take into consideration sample size.

#### Household Head’s Gender

**Fig 10:**
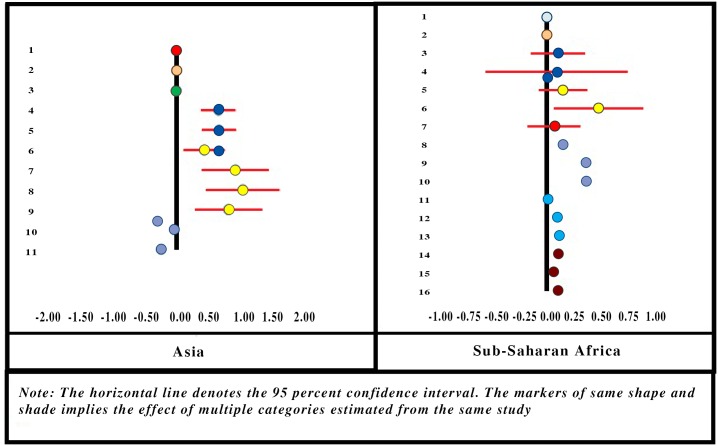
Forest-Plot for Household Head’s Gender.

Household head’s gender was reported by many authors as a factor influencing enrolment in CBHI (Fig 10). Two studies [63,73] involving 4 locations for the Asia region and 7 studies [21, 36, 44,49,50,61,68] involving 9 locations for the SSA region examined the variable in detail. A household with a female head in Asia was more likely to enroll in CBHI than one headed by a male. The result was uniform across the region although the absolute values of the effect size and its confidence intervals (CIs) varied. In the SSA region on the other hand the result was not uniform. Three [49, 61,68] of nine studies in the SSA region reported a positive association between the enrolment in CBHI and male-headed household, but the remaining studies reported almost zero, or highly negative association between the two. The summary effect is estimated as negative for both regions (-0.0505 for Asia, -0.3556 for SSA) and for the two regions combined (-0.359) (Table 1). However, the results are only indicative and not conclusive because the SE for the summary effect size could not be calculated.

In line with the results in meta-analysis, the vote count results show a similar pattern of relationship between female-headed households and enrolment—four-fifths of all quantitative studies show a positive association between the two.

### Results of Qualitative Synthesis (Thematic Synthesis)

**Fig 11:**
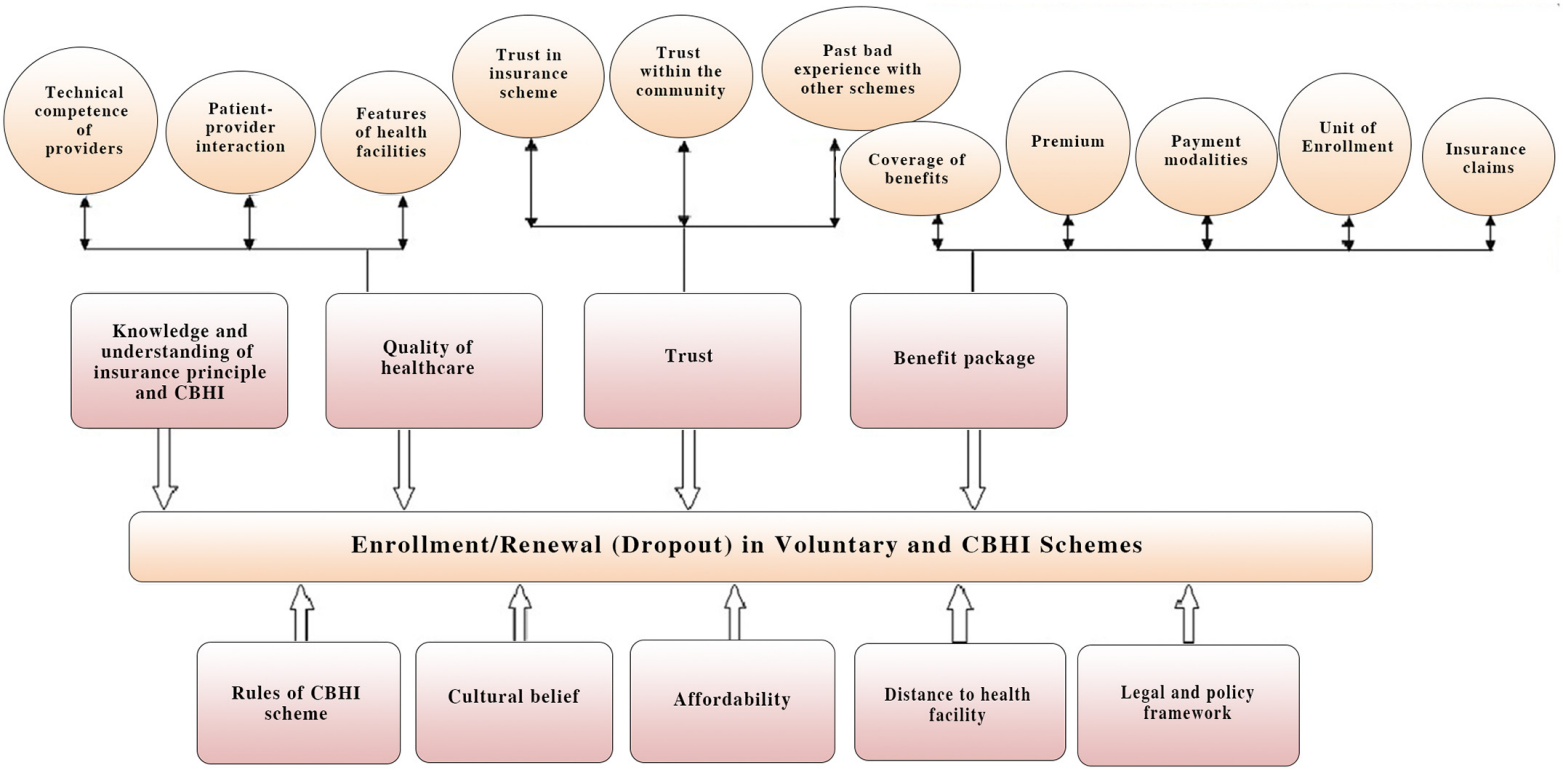
Themes and Subthemes Identified in CBHI Uptake and Dropout.

18 qualitative studies [25, 30–32, 33–35,38,48,60,62,64,67,70,71,72,75,76] have been used for the thematic synthesis; we identified nine major themes: knowledge and understanding of insurance principles and CBHI, quality of healthcare, trust, benefit package, rules of CBHI schemes, cultural beliefs, affordability, distance to health facility, and legal and policy framework (Fig 11 and S3b Table).

#### Theme 1—Knowledge and understanding of insurance principles and CBHI

This theme encompasses knowledge and understanding of prepayment, risk-pooling, redistribution of financial resources, CBHI managerial structure, responsibilities of different management levels, and CBHI benefits (including scheme features). Eleven studies reported knowledge and understanding of insurance principles and CBHI [30–32, 33,34,35,62,67,70,71,76].

Inadequate knowledge and understanding of insurance principles and CBHI were reported to be an obstacle to enrolment in 4 studies [31,33,48,62]. Limited understanding of the principles of CBHI by both beneficiaries and health providers and managers of CBHI was reported to be a barrier to enrolment in one study [30]. However, failure to understand the principles of CBHI did not explain low enrolment rates in one study [33]. A good understanding of the benefits of insurance was a facilitator of enrolment decision in 1 study [75]. Health insurance was poorly understood by some people as a form of “lotto” in 1 study [75]. Even in specific contexts where people had a broad understanding of insurance and CBHI, some legal terms (e.g., collaboration between CBHI and providers are regulated by a contract; CBHIs are managed following their by-laws) were not understood in 1 study [67] and some technical aspects of insurance (e.g., the risk of adverse selection; the advantages of large risk pool) were not fully understood in another study [33]. Although respondents comprehended the principle of insurance, they could not recall specific elements of scheme features including the CBHI managerial structure in 1 study [35]. In addition, one study reported poor knowledge and understanding of CBHI activities by key policy makers and health service managers [75,31]. Lack of clear understanding of insurance and prepayment mechanisms was reported to hamper scale-up of CBHI activities in 1 study [71]. Poor knowledge and understanding of CBHI was also reported to be a barrier to renewal in 2 studies [33,70].

#### Theme 2—Healthcare quality

The issues related to healthcare quality include three aspects: providers’ technical competence, patient-provider interactions and providers’ attitude and health facility features. Twelve studies reported these dimensions of quality of healthcare [25, 31,76,33,34,35, 48,64,67,71,72, 75]. Low healthcare quality was recognized by participants in one study [70] as one of the most important constraints to enrolment and membership renewal.

Healthcare providers’ lack of technical competence was reported to be a barrier to enrolment in two studies [33,67]. In one study it was argued that dropping out of the CBHI scheme could be explained by providers’ lack of technical expertise. [67]. The negative attitude of healthcare providers was reported to be a barrier to enrolment in five studies [31,33,48,71,75]. Participants expressed dissatisfaction with the negative attitude of providers toward patients in five studies [25, 34,67,72, 76]. The dissatisfaction was occasioned by long waiting queues, provider rudeness, preference given to cash-paying uninsured patients, and differential treatment depending on patient’s SES.

One study reported that 30 percent of members left a scheme because of providers’ negative behaviors [71]. Another study also reported that members dropped-out of the schemes due to providers’ rude behavior [40]. Four studies linked low enrolment to adverse features of a healthcare facility (dirty premises, unavailability of diagnostics, unavailability or shortage of prescribed medicines) [31,33,64,71]. In addition, two studies highlighted participants’ concerns about these poor features of healthcare facilities although they did not relate these features directly to either enrolment or renewal decisions [67,72].

#### Theme 3—Trust

This theme includes trust in insurance scheme management, trust within community and distrust associated with past bad experience of other schemes or collective arrangements. Fifteen studies commented on aspects relating to trust [25, 30,31, 33,34,35,48,62,64,67,70,71,72,75,76]

People’s trust in CBHI management was reported to be a facilitator of insurance enrolment decisions in four studies [25,34,62,67] and distrust a barrier to enrolment in five studies [30,35,71,72,75]. In addition, four studies highlighted the role of trust, although this was not in reference to specific enrolment/renewal decisions: poor involvement of the community in a hospital-based scheme [31]; lack of community participation in premium setting and managing funds [76]; criticism by respondents that the scheme failed to reach its objectives, defend its members, and keep its promises [33]; and low community participation an obstacle to sustaining the scheme [48]. In one study [70], members who renewed their membership had much stronger linkages with the scheme’s grassroots workers than did dropouts. Greater contact led to greater trust by scheme members.

Trust within the community was reported to be a facilitator of enrolment decision in one study [34], and also a facilitator of renewal decision in another study [70]. In both studies, participants highlighted that they joined because other villagers had joined or renewed, so they trusted the insurance scheme. Lack of trust within the community led to skepticism about who would manage the CBHI funds [35]. One study reported strong social capital or trust within the community, but limited trust outside the community and toward the government [64]. Lack of solidarity among community members was reported to be among the main reasons for non-enrolment in one study [67]. Previous bad experience and lack of trust in local financial organizations or other collective arrangements led communities to distrust the CBHI management and thus, not to enroll in the schemes in five studies [30,31,34,62,67]. The communities in such contexts were suspicious of the CBHI scheme and preferred ‘to wait and see whether CBHI would keep its promises before enrolling [67]. In fact, one study reported that past bad experience did not explain low enrolment because over time people gained confidence about scheme transparency and management’s trustworthiness [31].

#### Theme 4—Benefits package

This theme involves benefits package composition, premium, payment modalities, enrolment units and insurance claims. Twelve studies reported on aspects of benefits package [25, 31, 34,35, 38,60,64,67,70,71,75,76].

People’s dissatisfaction with the insurance benefits package was reported to be a major cause of low enrolment and membership renewal in three studies [31,48,71]. Exclusion of chronic diseases from the benefits package was reported to be a major weakness in four studies [31,48,64,72]. In one study, scheme dropouts suggested that the benefits package should cover out-patient care [70]. Participants voiced concerns about the provision of only second-level care (hospitalization) and lack of access to primary care at the healthcare centers in two studies [75,76]. Participants in four studies reported that the premium was fair and not too high [25,33–35]. One study reported that a high premium deterred people from joining the scheme [64]. The uninsured in another study reported that they did not join the scheme because they considered the premium too high [75]. However, in the same study, an equally high percentage of uninsured reported that they did not join due to an inappropriate registration period. Therefore, premium per se was not a decisive issue. As reported in two studies [48,76] participants criticized the flat-rate premium, in one case the same for children and adults [76] and in another case the same for both rich and poor, with no exemptions for the most vulnerable [48]. In fact, the participants in one study appreciated that CBHI has set different premiums for adults and children [35].

Paying the premium for the whole family at once was reported to be a major deterrent to enrolment in five studies [25, 34,35,71, 75] and a deterrent to renewal in one study [70]. Conversely, as reported in one study, payment by installment was an enabler for enrolment [30]. The timing of premium collections was noted as an enabler in one study: it was important for villagers to receive the CBHI card before being asked to pay for premiums [62]. In one study, participants criticized the inappropriate registration period and the fact that payment could not spread out over time [76].

Family/household enrolment was the norm in most of the schemes. Six studies reported that family enrolment for large families discouraged enrolment [25, 31,34,35,71,75]. In one study, enrolment was limited to four household members, which excluded larger families from the scheme and the coverage level was low [48]. One study found that a good claim experience could motivate renewal [70].

#### Theme 5—Rules of CBHI schemes

Arbitrary restrictions were found to inhibit CBHI enrolment and renewal [25,30,31,34,35,48]. Stringent rules imposed by management inhibited participation, both enrolment and renewal, four studies reported [25,30,31,48]. Many large families were reported to be excluded from some CBHI schemes due to restrictions on the number of persons that can be covered under a family membership (e.g., only up to four members can enroll) [48]. One study reported that unemployed or homeless persons are excluded from healthcare—most of the poorest segments of society [25]. Three studies reported difficulties in interesting the minimum number of people in CBHI membership so that enrolment could begin: 60 percent of a group or 100 families per village [30,31,48].

#### Theme 6—Cultural belief

Various sociocultural factors can either facilitate or act as barriers to enrolment in CBHI. One study reported that all the participants acknowledged that setting money aside for healthcare may be perceived as attracting diseases [34]. Some participants in that study further stated that when they save they do not talk about diseases. Prepayment before illness was associated with “inviting disease” in a study [31]. In another cultural context, participants reported that it is only when someone becomes sick that they ask the community to contribute financially to help a person [71]. In some cultures, women seek permission from their husbands to enroll [70]. See Box 2.

**Box 2. Non-Subscribers Views on Benefits Packages, CBHI Rules, Cultural Belief**

Benefits Package

“People with chronic diseases receive care from the doctor at the ambulatory; they get their drugs from the pharmacy where they often have to pay for the drugs. They can be a burden on their families; it is difficult to afford the drugs for many people. CBHI should cover these costs if possible.” [64]

“Some services are included and some are excluded. They have excluded some services because the money would not be enough to pay for them. I would like if one day, they could cover all services, but today it is good as it is, so that the insurance can have money till the end of the year.” [35]

Rules of CBHI Schemes

“If the CBHI people had said that I could divide the whole amounts in parts, I could have managed to enroll.’ [34]

“We did all we could to pay the entire premium. We looked for the money and we managed to find it. But for large families, this is very hard. It would be better if they could pay little by little. So, when they have some money, they turn that in. Then, when they find the rest, they pay again.” [35]

“The neediest people in our community especially the orphans, the disabled and the elderly still pay in the schemes. They have more health needs and should be excused.” [48]

“Rules should be change[d] so that those who don’t fall sick get something from the scheme.” [31]

“Why [can’t] the body of a subscriber who has died in hospital be transported to the villages?” [76]

Cultural Beliefs

“Paying before you fall sick is like buying a disease.” [31]

“It is the old people who say that if you keep an idea in your head, this thing will happen, but nowadays we do not think like this anymore.” [34]

“In our culture, it is only when someone becomes sick that we ask the community to contribute financially to help a person.” [71]

#### Theme 7—Affordability

Affordability involves people’s ability to pay the premium. All the studies noted that the poorest had been excluded from the CBHI scheme due to their inability to raise sufficient funds to pay the premium. Eleven studies commented on aspects of affordability [25,30,31, 33,34,35,62,64,70,75,76]. Lack of financial means was the most common reason for people not enrolling in CBHI schemes, mentioned in 11 studies [25, 30,31, 33–35, 62,64,70,75,76]. Lack of affordability was also a reason for people dropping out of the scheme [65]. However, one study reported that lack of money was not a major issue for renewal decision [70]. While lack of money was a common response for not being able to join the scheme, especially for poor households, many studies noted that the real issue was the unavailability of funds at the time when payment was collected [25, 34,70,75].

#### Theme 8—Distance from health facility

Travel and transport, too, can act as facilitators or barriers in accessing healthcare at the facilities contracted by the CBHI scheme [25,27,31,34,64,71]. One study reported that 25 percent of the unenrolled could not join the scheme because there was no facility nearby [25]. Long distance from the communities to a healthcare facility was reported to be an obstacle to enrolment in one study [27]. Two studies reported that high transport cost was a reason for low enrolment [64,71].

#### Theme 9—Legal and policy framework

Various legal and policy frameworks with enrolment/renewal decisions were evident. Nine studies discussed this theme affecting uptake in CBHI [30,32,48,62,64,67,70,71,72]. Four studies highlighted the absence of a coherent legal, regulatory and policy framework as a direct obstacle to maximizing CBHI membership [62,67,70,71]. One study reported that many insured members had dropped out of the CBHI schemes as they were skeptical about CBHI operations without appropriate legislative backup from the government [48]. The importance of legal and policy framework was discussed in the context of the sustainability of CBHI schemes in three studies [32,67,72].

Box 3 contains testimonials on the above themes.

**Box 3. Barriers to Health Insurance: Affordability, Distance, Inadequate Legal and Policy Framework**

Affordability-

"We are not refusing to pay, but we cannot afford to" [76]

“I wanted to enroll, but I did not find the means, maybe next year….” [34]

"The only reason for not joining is money. If we had money we would join, but our village is the poorest of the poor.” [64]

"If people cannot afford to pay now, how will they afford to pay if you increase the premiums?" [64]

Why should it be the same premium for everyone, when there are different charges for adults and children at the health centre and the hospital? [76]

"Out here in the countryside, the availability of money poses a problem……. we, the farmers, have money after the harvest, but by the time the rainy season arrives, we have nothing left in our hand and out here you cannot find where to borrow money. [35]

Distance to Health Facility-

“It was expensive for me to travel 27 Km to and from Ishaka hospital.” [31]

“Transport is a problem. Our village is isolated, and the road is not good. In winter it is very difficult to even get to Vayk.” [64]

“If there was a doctor in our village, more people would enroll…. To have a doctor right at your side would encourage many to enter.” [34]

Quality of Care

"The care given to us at the hospital is good but we cannot afford joining the scheme." [30]

Legal and Policy Framework-

“For me, the solution is that [health insurance] becomes obligatory and that there’s a real constraint to enroll. Without this, MHOs [mutual health organizations] will not survive.” [71]

“It should be feasible to roll-out CBHI schemes nationally, but technical and managerial oversight would be needed. There is no role for the government in this; it should be provided by NGOs.” [64]

“No policy yet but CHI [community health insurance] is a component of the ministerial policy statement.” [30]

“Health is something that everyone needs to maintain, and therefore CHI has a place in Uganda. Let us start with national policies facilitating CHI…. Regulations are very important and gradual implementation is needed.” [32]

## Discussion

The systematic review of the literature set out to provide evidence on demand- and supply-side factors affecting uptake of CBHI memberships in LMIC. The evidence, originating from 54 rigorously screened articles, is discussed here based on the meta-analysis and a thematic synthesis.

### Demand-Side Factors

On the demand side, the strongest factor affecting uptake of CBHI is the socioeconomic status (SES) of the household. The results of meta-analysis confirm a positive association of SES with enrolment (summary effect is significantly higher than zero), regardless of whether the SES is expressed in terms of income, expenditure or asset ownership. The importance of this factor is strengthened by the obverse evidence from the thematic synthesis that low affordability was one of the barriers to enrolment or renewing membership. Interestingly, lack of affordability was not synonymous with low income in all cases, but could also mean inability to pay the annual premium upfront (i.e., compliance with administrative modalities) or the inability to pay at the time premiums were collected (e.g., before the harvest season).

In Asia, the second most prominent demand-side factor affecting enrolment is the marital status of the household head (but significance could not be confirmed as some authors did not report the SE of their estimates). It emerged clearly from the meta-analysis that Asian married household heads were more disposed to enroll, and that this factor was not equally weighty in Africa. Female-headed households (in Asia) were more likely to enroll than male-headed households (source: meta-analysis), but also more likely to drop out. This may reflect possible dynamics toward female household heads within schemes. Neither marital status nor gender of the household head emerged as factors in the thematic synthesis.

Education of the household head, another individual attribute, was positively correlated with both enrolment and renewal decisions in CBHI schemes (meta-analysis), and this summary effect was significantly higher than zero. The thematic synthesis brought to light that knowledge and understanding of insurance principles and the functioning of the CBHI facilitated both enrolment and renewal decisions. In communities where literacy is low and information scarce, low enrolment and renewal decisions may be related to people’s low understanding of CBHI. While education can be counted as a demand-side factor, efforts made by insurance companies to explain their value proposition reflect supply-side factors to acquire potential clients that are free to enroll or not. Low enrolment linked to low familiarity with CBHI operations may be counted as a supply-side factor.

Another demand-side factor that enables enrolment to CBHI (confirmed through meta-analysis) is prevalence of chronic illness in the household (caveat: statistical significance of the summary effect could not be established as some studies did not provide information on SE). The importance of this factor must be considered in the context that such diseases are on the rise in all LMIC, and augmented by the increase of life expectancy. The household head’s age was confirmed to be a facilitator of enrolment (in meta-analysis but not in the thematic synthesis) but the presence of elderly persons in the household was a barrier to enrolment. These results might at first seem contradictory, but are really quite complementary. It is self-explanatory why household head age is positively associated with enrolments; and it seems also self-explanatory that when elderly people cannot expect to obtain services they want from their CBHI scheme they obviously would not see reason to enroll. A similar effect could be expected of chronically ill: higher propensity to enroll when the scheme covers at least part of their higher costs, and lower enrolments when schemes do not cover their needs. These factors have a supply-side angle in that responsibility for benefits package design is vested with schemes. The benefits limitations that CBHI schemes observe arise from the need to remain sustainable even as they keep premiums low (a clear plus point for enrolments) and cannot normally rely on subsidy income.

Finally, household size was positively associated with enrolment in the meta-analysis (and the summary effect was significantly higher than zero) but in the thematic synthesis was flagged as a barrier to enrolment, when large households could not pay premiums for all their members at one go. The seemingly conflicting information may be due to the inclusion of different pricing practices of some CBHI in the quantitative analysis, for example, premium discount to large households. The meta-analysis suggests that household size was a facilitator of renewal as well. None of the qualitative studies looked at this aspect.

### Supply-Side Factors

We now come to supply-side factors. The thematic synthesis found that *trust in the scheme management* was a significant enabler of enrolment. This aspect was not considered in the meta-analysis, but the closely related *trust in insurance scheme* was, and found to facilitate renewal decisions. Two closely related themes had similar effects: when the rules of CBHI schemes were perceived as rigid, and when people felt there was lack of clarity about the legal or policy framework, they were less inclined to enroll and renew. All these factors show that, when a scheme is trusted, considered accommodating, and enjoys support from policymakers, it attracts higher enrolments.

Other supply-side factors that emerged in the thematic synthesis (and not at all considered in the meta-analysis) include the self-explanatory situation that when healthcare services were considered of good quality they were an important enabler of enrolment and renewal decisions. And distance from residence to healthcare facility was found to be an obstacle to enrolment in the thematic synthesis.

This systematic review deals with voluntary and contributory enrolment and renewal; SES was shown to be an enabling factor, but as people’s SES improves, they may consume more of everything and also of healthcare services, beyond what the CBHI covers. Thus, just as there might be a positive association between enrolment and SES, there could be a negative association between increased SES and renewal, if the gap grows between what a household can afford and what the CBHI offers. This was established through the meta-analysis. However, the net effect would be influenced by other operational practices of the CBHI, such as its track record in paying claims promptly and correctly. The thematic synthesis confirmed that positive experience with how the CBHI settled claims was an important enabler of renewal decision.

### Conclusions and Policy Implications

This systematic review examined the evidence of factors affecting voluntary uptake of community-based health insurance schemes in low- and middle-income countries, with a view to including CBHI as part of a strategy to extend the outreach of social protection. The evidence shows that certain factors affecting voluntary uptake in CBHI are basically demand-driven, while others are driven by conditions controlled by the supply-side or by policy.

The first major insight relates to demand-side factors positively affecting enrolment in CBHI, as well as renewals: Education, age, female household heads, and the socioeconomic status of households are all factors that positively affect enrolments. Moreover, when individuals understand insurance principles in general, and how their CBHI functions, they are more likely to enroll; when people have a positive claims experience, they are more likely to renew. A higher prevalence of chronic conditions enhances the likelihood of enrolling; the perception that healthcare is of good quality and nearby acts as a factor enhancing enrolment, and the perception that services are distant or deficient leads to lower enrolments. All this reflects common-sense. Moreover, the finding that having to pay annual premiums upfront creates financial stress which was not limited to poor households suggests that this problem affecting uptake and renewal in CBHI is inherently solvable, as it originates mainly from administrative convenience, and could be resolved or at least significantly reduced, when arrangements are put in place to spread premium payment over the entire coverage period. Such arrangements are commonly in place in social health insurance, and could be applied at CBHI level as well, either through regulation and/or through suitable arrangements with local MFIs.

The second insight is that many supply side issues that are within the control of CBHI schemes and related organizations can be adjusted to enhance enrolment and renewals as well. Trust in the *scheme management* and trust in the *insurance scheme* emerged as significant enablers of enrolment. Schemes viewed by the target population as applying rules rigidly (i.e., unfairly) attract fewer enrolments. Stated simply, the evidence indicates that even community-based schemes must maintain a positive image, or risk alienation from their catchment communities.

The third insight is that clarity about the legal or policy framework also acts as a factor influencing enrolments, and lack of clarity dampens uptake. The evidence discovered through this systematic review offers for the first time a clear indication that the prospective members of CBHIs harbor two balancing expectations: on the one hand, an expectation (from governments and the development community) to recognize that CBHI schemes function within a legitimate framework, and by implication that CBHIs are fulfilling a desirable social role; and on the other hand, an expectation from the CBHI to operate a client-centric scheme at local level, with measures to ensure clients that the insurance contract can and will be enforced fairly, rather than being complementary to, or dependent on other schemes or vertical programs or external governance. This conclusion differs fundamentally from that of Ekman [12] who claimed that CBHI were at best *complementary to other more effective systems of health financing*. There is no contradiction between the wish of the population to take advantage of benefits from a local scheme, while simultaneously expecting the authorities to provide a coherent framing to recognize such local scheme as legitimate. This very pragmatic nexus suggests that people recognize that they must pay for health insurance, and prefer localized operations through which they stand a better chance to gain welfare by being involved not merely in paying but also in priority-setting and in scheme governance. This is also fundamentally different from conclusions reached by Adebayo et al [14] that the challenges of CBHI are captured by *lack of funds*, *poor quality of care*, *and lack of trust*, i.e. three deficiencies reflecting unmet expectations from others: funders, providers and administrators. Our analysis has shown that the target population has expectations to influence priority-setting or governance when they participate voluntarily in CBHI schemes. This expectation of the target population suggests that governments—especially in low income countries—could leverage people’s willingness to exercise voluntary and contributory enrolment in a community-based health insurance, by acting as regulators even when they cannot act as funders and providers. The limitations of low-income governments to act as funders and providers of services are well known and there are no easy solutions at hand today; our conclusions that people at the base of the pyramid are more likely to enroll in CBHI on a voluntary basis when governments formulate an enabling environment for the development and expansion of CBHI offer an important unexplored opportunity to facilitate the growth of this form of health financing from niche to mass.

## Limitations

This research report was limited by the number, quality and themes of the published literature. Thus, the filtering process could deal only with the quality of articles, but not with the meager number or with the themes that were chosen by the various authors. As the largest number of studies dealt with CBHI schemes in Sub-Saharan Africa, the conclusions might be more relevant for the African context. This is particularly limiting in the case of a nascent activity like CBHI, where the history of publications spans barely a decade and relies mainly on the African experience but where much of the growth in activities now occurs in Asia. On the quality of publications, other than the 8 articles that were considered unsuitable and excluded, all the studies retained for full text analysis were then considered as equal in terms of quality [26] suggested that this could possibly bias the pooled results. A number of authors did not report the SEs or their estimates. Hence, we applied weights proportional to the sample size (instead of inverse of the variance, which is the standard practice of meta-analysis) while estimating the summary effect. We also recognize the somewhat weak internal validity of certain studies due to flaws in study methodology. Although we attempted to account for this utilizing the PRISMA protocol for the search strategy, this corrective approach has its limitations. Sample sizes for some of the studies were large [21] and sensitivity analysis shows that it does have some influence on the estimated summary effect. For some variables, the results are only indicative and not conclusive as the SE was not reported in all studies, and consequently the summary effect size could not be calculated.

## Supporting Information

S1 Table PRISMA Checklist. (DOCX)

S2 Table Quality Assessment of Included Studies. (DOCX)

S3 Table Characteristics of Included Studies (Quantitative; Qualitative; Mixed-method papers). (DOCX)

S1 Text Search Strategy. (DOCX)

S2 Text Data Extraction Sheet. (DOCX)
