# Supplementary material for: What Factors Affect Voluntary Uptake of Community-Based Health Insurance Schemes in Low- and Middle-Income Countries? A Systematic Review and Meta-Analysis
Source: PLoS One. 2016 Aug 31;11(8):e0160479. doi: 10.1371/journal.pone.0160479 (PMC5006971; doi:10.1371/journal.pone.0160479)
Supplement: S2 Table — (DOCX) [file pone.0160479.s002.docx]

## S2 Table: Quality Assessment of Included Studies

# Quantitative Studies

|  | Aggarwal (2010) | Akotey (2011) | De Allegri (2006c) | Bendig (2011) | Nsiah-Boateng (2013) | Chankova (2008) | Donfouet (2012) | Dong (2005) | Dong (2009) | Dror (2010) | Eckhardt (2011) | Msuya (2004) | Fonta (2010) |
| --- | --- | --- | --- | --- | --- | --- | --- | --- | --- | --- | --- | --- | --- |
| 1. Is the research aim clearly stated? | Yes | Yes | Yes | Yes | Yes | Yes | Yes | Yes | Yes | Yes | Yes | Yes | Yes |
| 2. Description of the context? | Yes | Yes | Yes | Yes | Yes | Yes | Yes | Yes | Yes | Yes | Yes | Yes | Yes |
| 3. Description of the sampling procedures? | Yes | Yes | Yes | Yes | Yes | Yes | Yes | Yes | Yes | Yes | Yes | Yes | Yes |
| 4. Are sample characteristics sufficiently reported? (sample size, location, and at least one additional characteristic) | Yes | Yes | Yes | Yes | Yes | Yes | Yes | Yes | Yes | Yes | Yes | Yes | Yes |
| 5. Is it clear how the data were collected (eg: for interviews, is there an indication of how interviews were conducted? | Yes | Yes | Yes | Yes | Yes | Yes | Yes | Yes | Yes | Yes | Yes | Yes | Yes |
| 6. Methods of recording of data reported? | No | No | Yes | No | No | Yes | No | Yes | No | Yes | Yes | Yes | No |
| 7. Methods of analysis explicitly stated? | Yes | Yes | Yes | Yes | Yes | Yes | Yes | Yes | Yes | Yes | Yes | Yes | Yes |
| 8.  Is there a clear link to relevant literature/theoretical framework? | Yes | Yes | Yes | Yes | Yes | Yes | Yes | Yes | Yes | Yes | Yes | Yes | Yes |
| 9.  Is the design appropriate to answer the research question? | Yes | Yes | Yes | Yes | Yes | Yes | Yes | Yes | Yes | Yes | Yes | Yes | Yes |
| 10. Was the sampling strategy appropriate to the aims of the research? | Yes | No | No | Yes | Yes | Yes | Yes | Yes | Yes | Yes | Yes | Yes | Yes |
| 11. Were the data collected in a way that addressed the research issue? | Yes | Yes | Yes | Yes | Yes | Yes | Yes | Yes | Yes | Yes | Yes | Yes | Yes |
| 12.Yes Is there a detailed description of the analysis process? | Yes | Yes | Yes | Yes | Yes | Yes | Yes | Yes | Yes | Yes | Yes | Yes | Yes |
| 12.2. Does the data support the findings? | Yes | Yes | Yes | Yes | Yes | Yes | Yes | Yes | Yes | Yes | Yes | Yes | Yes |
| 12.3. If the findings are based on quantitative analysis of survey data, then are multivariate techniques used to control for potential confounding variable? | Yes | Yes | Yes | Yes | No | Yes | Yes | Yes | Yes | No | No | Yes | Yes |
| 16. Does the paper discuss ethical considerations related to the research? | No | No | Yes | No | Yes | Yes | No | No | No | No | Yes | No | No |

# Quantitative Studies (continued)

|  | Gnawali (2009) | Gumber (2001) | Wang H.(2005) | Ito (2010) | Lammers (2010) | Jutting (2003) | Kuwawenaruwa (2011) | Mathiyazhagn (1998) | Msuya (2007) | Noubiap (2013) | Onwujekwe (2009) | Onwujekwe (2011) |
| --- | --- | --- | --- | --- | --- | --- | --- | --- | --- | --- | --- | --- |
| 1. Is the research aim clearly stated? | Yes | Yes | Yes | Yes | Yes | Yes | Yes | Yes | Yes | Yes | Yes | Yes |
| 2. Description of the context? | Yes | Yes | Yes | Yes | Yes | Yes | Yes | Yes | Yes | Yes | Yes | Yes |
| 3. Description of the sampling procedures? | Yes | Yes | Yes | Yes | Yes | Yes | Yes | Yes | Yes | Yes | Yes | Yes |
| 4. Are sample characteristics sufficiently reported? (sample size, location, and at least one additional characteristic) | Yes | Yes | Yes | Yes | Yes | Yes | Yes | Yes | Yes | Yes | Yes | Yes |
| 5. Is it clear how the data were collected (eg: for interviews, is there an indication of how interviews were conducted? | Yes | Yes | Yes | Yes | Yes | Yes | Yes | Yes | Yes | Yes | Yes | Yes |
| 6. Methods of recording of data reported? | Yes | No | No | No | No | Yes | Yes | No | No | Yes | Yes | Yes |
| 7. Methods of analysis explicitly stated? | Yes | Yes | Yes | Yes | Yes | Yes | Yes | Yes | Yes | Yes | Yes | Yes |
| 8.  Is there a clear link to relevant literature/theoretical framework? | Yes | Yes | Yes | Yes | Yes | Yes | Yes | Yes | Yes | Yes | Yes | Yes |
| 9.  Is the design appropriate to answer the research question? | Yes | Yes | Yes | Yes | Yes | Yes | Yes | Yes | Yes | No | Yes | Yes |
| 10. Was the sampling strategy appropriate to the aims of the research? | Yes | No | Yes | Yes | Yes | Yes | Yes | Yes | Yes | No | Yes | Yes |
| 11. Were the data collected in a way that addressed the research issue? | Yes | Yes | Yes | Yes | Yes | Yes | Yes | Yes | Yes | No | Yes | Yes |
| 12.Yes Is there a detailed description of the analysis process? | Yes | Yes | Yes | Yes | Yes | Yes | Yes | Yes | Yes | Yes | Yes | Yes |
| 12.2. Does the data support the findings? | Yes | Yes | Yes | Yes | Yes | Yes | Yes | Yes | Yes | Yes | Yes | Yes |
| 12.3. If the findings are based on quantitative analysis of survey data, then are multivariate techniques used to control for potential confounding variable? | Yes | Yes | Yes | Yes | Yes | Yes | Yes | Yes | Yes | No | No | Yes |
| 16. Does the paper discuss ethical considerations related to the research? | Yes | No | Yes | Yes | Yes | Yes | No | No | No | Yes | Yes | No |

# Quantitative Studies (continued)

|  | Oriakhi (2012) | Panda (2013) | Mladovsky (2014) | Bhatt (2006) | Rao (2009) | Schneider (2001) | Shafie (2013) | Zhang (2006) |
| --- | --- | --- | --- | --- | --- | --- | --- | --- |
| 1. Is the research aim clearly stated? | Yes | Yes | Yes | Yes | Yes | Yes | Yes | Yes |
| 2. Description of the context? | Yes | Yes | Yes | Yes | Yes | Yes | Yes | Yes |
| 3. Description of the sampling procedures? | Yes | Yes | Yes | Yes | Yes | Yes | Yes | Yes |
| 4. Are sample characteristics sufficiently reported? (sample size, location, and at least one additional characteristic) | Yes | Yes | Yes | Yes | Yes | Yes | Yes | Yes |
| 5. Is it clear how the data were collected (eg: for interviews, is there an indication of how interviews were conducted? | Yes | Yes | Yes | Yes | Yes | Yes | Yes | Yes |
| 6. Methods of recording of data reported? | Yes | Yes | No | Yes | No | Yes | Yes | Yes |
| 7. Methods of analysis explicitly stated? | Yes | Yes | Yes | Yes | Yes | Yes | Yes | Yes |
| 8.  Is there a clear link to relevant literature/theoretical framework? | Yes | Yes | Yes | Yes | Yes | Yes | Yes | Yes |
| 9.  Is the design appropriate to answer the research question? | Yes | Yes | Yes | Yes | Yes | Yes | Yes | Yes |
| 10. Was the sampling strategy appropriate to the aims of the research? | Yes | Yes | No | Yes | Yes | Yes | Yes | Yes |
| 11. Were the data collected in a way that addressed the research issue? | Yes | Yes | No | Yes | Yes | Yes | Yes | Yes |
| 12.1. Is there a detailed description of the analysis process? | Yes | Yes | Yes | Yes | Yes | Yes | Yes | Yes |
| 12.2. Does the data support the findings? | Yes | Yes | Yes | Yes | Yes | Yes | Yes | Yes |
| 12.3. If the findings are based on quantitative analysis of survey data, then are multivariate techniques used to control for potential confounding variable? | Yes | Yes | Yes | Yes | No | Yes | Yes | Yes |
| 16. Does the paper discuss ethical considerations related to the research? | No | Yes | Yes | No | No | No | No | No |

*Source*:

Waddington H, Snilstveit B, Hombrados GJ, Vojtkova M, Anderson J, White H (2012) Protocol: Farmer Field Schools for Improving Farming Practices and Farmer Outcomes in Low- and Middle-income Countries: *A Systematic Review.* Available at [*http://campbellcollaboration.org/lib/project/203/*](http://campbellcollaboration.org/lib/project/203/).

*Note*: Questions are answered as *Yes* (Denoted by 1) and *No* (Denoted by 0).

Question No 13-15 is not given in this table because they are not relevant.

# Qualitative Studies

|  | Allegri (2006a) | Allegri (2006b) | Atim (2000) | Basaza (2007) | Basaza (2008) | Criel (1998) | Criel (2003) | Kyomugisha (2009) | Poletti (2007) | Schneider (2005) | Tremblay (2012) |
| --- | --- | --- | --- | --- | --- | --- | --- | --- | --- | --- | --- |
| 1. Is the research aim clearly stated? | Yes | Yes | Yes | Yes | Yes | Yes | Yes | Yes | Yes | Yes | Yes |
| 2. Description of the context? | Yes | Yes | Yes | Yes | Yes | Yes | Yes | Yes | Yes | Yes | Yes |
| 3. Description of the sampling procedures? | Yes | Yes | Yes | Yes | Yes | Yes | Yes | Yes | Yes | Yes | Yes |
| 4. Are sample characteristics sufficiently reported? (sample size, location, and at least one additional characteristic) | Yes | Yes | Yes | Yes | Yes | Yes | Yes | Yes | Yes | Yes | Yes |
| 5. Is it clear how the data were collected (eg: for interviews, is there an indication of how interviews were conducted? | Yes | Yes | Yes | Yes | Yes | Yes | Yes | Yes | Yes | Yes | Yes |
| 6. Methods of recording of data reported? | Yes | Yes | Yes | Yes | Yes | No | Yes | Yes | Yes | Yes | Yes |
| 7. Methods of analysis explicitly stated? | Yes | Yes | Yes | Yes | Yes | No | Yes | Yes | Yes | Yes | Yes |
| 8. Was there clear statement of aims of the research? | Yes | Yes | Yes | Yes | Yes | Yes | Yes | Yes | Yes | Yes | Yes |
| 9. Is a qualitative methodology appropriate? | Yes | Yes | Yes | Yes | Yes | Yes | Yes | Yes | Yes | Yes | Yes |
| 10. Was the research design appropriate to address the aims of the research? | Yes | Yes | Yes | Yes | Yes | Yes | Yes | Yes | Yes | Yes | Yes |
| 11. Was the recruitment strategy appropriate to the aims of the research? | Yes | Yes | Yes | Yes | Yes | Yes | No | Yes | Yes | Yes | Yes |
| 12. Were the data collected in a way that addressed the research issue? | Yes | Yes | Yes | Yes | Yes | Yes | Yes | Yes | Yes | Yes | Yes |
| 13. Has the relationship between researcher and participants been adequately considered? | Yes | No | Yes | No | Yes | No | Yes | No | No | Yes | Yes |
| 14. Have ethical issuers been taken into consideration? | Yes | No | No | Yes | No | No | No | Yes | No | No | Yes |
| 15. If there is an in depth description of the analysis process? | Yes | Yes | Yes | Yes | Yes | Yes | Yes | Yes | Yes | Yes | Yes |
| 16. Is there a clear statement of findings? IF the findings are explicit? | Yes | Yes | Yes | Yes | Yes | Yes | Yes | Yes | Yes | Yes | Yes |
| 17. How valuable is the research? If the researcher discusses the contribution of the study to the existing knowledge or understanding? | Yes | Yes | Yes | Yes | Yes | Yes | Yes | Yes | Yes | Yes | Yes |

*Source*:

Question No. (1-7) from Waddington H, Snilstveit B, Hombrados GJ, Vojtkova M, Anderson J, White H (2012) Protocol: Farmer Field Schools for Improving Farming Practices and Farmer Outcomes in Low- and Middle-income Countries: *A Systematic Review.* Available at [*http://campbellcollaboration.org/lib/project/203/*](http://campbellcollaboration.org/lib/project/203/)

Question No. (8-17) from Critical Appraisal Skills Programme (CASP). (2006). *10 questions to help you make sense of qualitative research.* Public Health Resource Unit: England. [http://www.casp-uk.net/#!casp-tools-checklists/cYes8f8](http://www.casp-uk.net/#!casp-tools-checklists/c18f8)

*Note*: Questions are answered as *Yes* (Denoted by 1) and *No* (Denoted by 0).

# Mixed Method Studies

|  | Dong (2004) | Onwujekwe (2010) | Uzochukwu (2009) |
| --- | --- | --- | --- |
| 1. Is the research aim clearly stated? | Yes | Yes | Yes |
| 2. Description of the context? | Yes | Yes | Yes |
| 3. Description of the sampling procedures? | Yes | Yes | Yes |
| 4. Are sample characteristics sufficiently reported? (sample size, location, and at least one additional characteristic) | Yes | Yes | Yes |
| 5. Is it clear how the data were collected (eg: for interviews, is there an indication of how interviews were conducted? | Yes | Yes | Yes |
| 6. Methods of recording of data reported? | No | Yes | Yes |
| 7. Methods of analysis explicitly stated? | Yes | Yes | Yes |
| Quantitative |  |  |  |
| 8. Is there a clear link to relevant literature/theoretical framework? | Yes | Yes | Yes |
| 9. Is the design appropriate to answer the research question? | Yes | Yes | Yes |
| 10. Was the sampling strategy appropriate to the aims of the research? | Yes | Yes | Yes |
| 11. Were the data collected in a way that addressed the research issue? | Yes | Yes | Yes |
| 12.1. Is there a detailed description of the analysis process? | Yes | Yes | Yes |
| 12.2. Does the data support the findings? | Yes | Yes | Yes |
| 12.3. If the findings are based on quantitative analysis of survey data, then are multivariate techniques used to control for potential confounding variable? | No | No | No |
| 16. Does the paper discuss ethical considerations related to the research? | No | Yes | Yes |
| Qualitative |  |  |  |
| 8. Was there clear statement of aims of the research? | Yes | Yes | Yes |
| 9. Is a qualitative methodology appropriate? | Yes | Yes | Yes |
| 10. Was the research design appropriate to address the aims of the research? | Yes | Yes | Yes |
| 11. Was the recruitment strategy appropriate to the aims of the research? | Yes | Yes | Yes |
| 12. Were the data collected in a way that addressed the research issue? | Yes | Yes | No |
| 13. Has the relationship between the researcher and the participants adequately considered? | Yes | Yes | Yes |
| 14. Have ethical issues been taken into consideration? | No | Yes | Yes |
| 15. If there is an in depth description of the analysis process? | Yes | Yes | Yes |
| 16. Is there a clear statement of findings? IF the findings are explicit? | Yes | Yes | Yes |
| 17. How valuable is the research? If the researcher discusses the contribution of the study to the existing knowledge or understanding? | Yes | Yes | Yes |

*Source*:

Question No. (1-16) from Waddington H, Snilstveit B, Hombrados GJ, Vojtkova M, Anderson J, White H (2012) Protocol: Farmer Field Schools for Improving Farming Practices and Farmer Outcomes in Low- and Middle-income Countries: *A Systematic Review.* Available at [*http://campbellcollaboration.org/lib/project/203/*](http://campbellcollaboration.org/lib/project/203/).

Question No. (8-17) from Critical Appraisal Skills Programme (CASP). (2006). *10 questions to help you make sense of qualitative research.* Public Health Resource Unit: England. [http://www.casp-uk.net/#!casp-tools-checklists/cYes8f8](http://www.casp-uk.net/#!casp-tools-checklists/c18f8)

*Note*: Questions are answered as *Yes* (Denoted by 1) and *No* (Denoted by 0).

Question No 13-15 is not given in the quantitative section of Table because these are not appropriate for the current table.

# Cohort Studies

|  |  | Quantitative | | Qualitative | Mixed Method | | |
| --- | --- | --- | --- | --- | --- | --- | --- |
|  |  | Liu(2013) | Ranson (2001) | Basaza (2010) | Alatinga(2011) | Ozawa (2009) | Sinha (2006) |
|  | Is the research aim clearly stated? | Yes | Yes | Yes | Yes | Yes | Yes |
|  | Description of the context? | Yes | Yes | Yes | Yes | Yes | Yes |
|  | Description of the sampling procedures? | Yes | Yes | Yes | Yes | Yes | Yes |
|  | Are sample characteristics sufficiently reported? (sample size, location, and at least one additional characteristic) | Yes | Yes | Yes | Yes | Yes | Yes |
|  | Is it clear how the data were collected (eg: for interviews, is there an indication of how interviews were conducted? | Yes | Yes | Yes | Yes | Yes | Yes |
|  | Methods of recording of data reported? | Yes | Yes | Yes | Yes | Yes | No |
|  | Methods of analysis explicitly stated? | Yes | Yes | Yes | Yes | Yes | Yes |
|  | Did the study address a clearly focused issue? | No | Yes | Yes | Yes | Yes | Yes |
|  | Was the cohort recruited in an acceptable way? | No | Yes | Yes | Yes | Yes | Yes |
|  | Was the exposure accurately measured to minimise bias? | Yes | Yes | No | Yes | Yes | No |
|  | Was the outcome accurately measured to minimise bias? | Yes | Yes | No | Yes | Yes | No |
|  | Have the authors identified all important confounding factors? | Yes | Yes | Yes | Yes | Yes | Yes |
|  | Have they taken account of the confounding factors in the design and/or analysis? | Yes | Yes | Yes | Yes | Yes | Yes |
|  | Was the follow up of subjects complete enough? | Yes | Yes | Yes | Yes | Yes | Yes |
|  | Was the follow up of subjects long enough? | Yes | Yes | Yes | Yes | Yes | Yes |
|  | What are the results of this study? | Yes | Yes | Yes | Yes | Yes | Yes |
|  | How precise are the results? | Yes | Yes | Yes | Yes | Yes | Yes |
|  | Do you believe the results? | Yes | Yes | Yes | Yes | Yes | Yes |
|  | Can the results be applied to the local population? | Yes | Yes | Yes | Yes | Yes | Yes |
|  | Do the results of this study fit with other available evidence? | Yes | Yes | Yes | Yes | Yes | Yes |
|  | What are the implications of this study for practice? | Yes | Yes | Yes | Yes | Yes | Yes |

*Source:*

Question No. (1-7) from Waddington H, Snilstveit B, Hombrados GJ, Vojtkova M, Anderson J, White H (2012) Protocol: Farmer Field Schools for Improving Farming Practices and Farmer Outcomes in Low- and Middle-income Countries: *A Systematic Review.* Available at [*http://campbellcollaboration.org/lib/project/203/*](http://campbellcollaboration.org/lib/project/203/).

Question No. (8-21) from Critical Appraisal Skills Programme (CASP). (2013). *Yes2 questions to help you make sense of cohort study.* Public Health Resource Unit: England. [http://www.casp-uk.net/#!casp-tools-checklists/cYes8f8](http://www.casp-uk.net/#!casp-tools-checklists/c18f8);

*Note*: Questions are answered as *Yes* (Denoted by 1) and *No* (Denoted by 0).

# RCT Study

|  |  | Bonan (2012) |
| --- | --- | --- |
| 1 | Is the research aim clearly stated? | Yes |
| 2 | Description of the context? | Yes |
| 3 | Description of the sampling procedures? | Yes |
| 4 | Are sample characteristics sufficiently reported? (sample size, location, and at least one additional characteristic) | Yes |
| 5 | Is it clear how the data were collected (eg: for interviews, is there an indication of how interviews were conducted? | Yes |
| 6 | Methods of recording of data reported? | Yes |
| 7 | Methods of analysis explicitly stated? | Yes |
| 8 | Random sequence generation: selection bias due to inadequate generation of a randomised sequence. | Low Risk |
| 9 | Allocation concealment: selection bias due to inadequate concealment of allocations prior to assignment. | Low Risk |
| 10 | Performance bias: due to knowledge of the allocated interventions by participants and personnel during the study | Low Risk |
| 11 | Detection bias: due to knowledge of the allocated interventions by outcome assessors. | Low Risk |
| 12 | Attrition Bias: due to amount, nature or handling of incomplete outcome data | Low Risk |
| 13 | Reporting bias: due to selective outcome reporting. | Low Risk |
| 14 | Other bias: due to problems not covered anywhere else. | Unclear Risk |

*Source:*

Question No. (1-7) from Waddington H, Snilstveit B, Hombrados GJ, Vojtkova M, Anderson J, White H (2012) Protocol: Farmer Field Schools for Improving Farming Practices and Farmer Outcomes in Low- and Middle-income Countries: *A Systematic Review.* Available at [*http://campbellcollaboration.org/lib/project/203/*](http://campbellcollaboration.org/lib/project/203/).

Question No. (8-14) from Higgins, J., & Green, S. (Eds.). (2011). *Cochrane handbook for systematic reviews of interventions. (Version 5.0.2, updated September 2009).* The Cochrane Collaboration. Available at [www.cochrane-handbook.org](http://www.cochrane-handbook.org)

*Note*: Questions are answered as *Yes* (Denoted by 1) and *No* (Denoted by 0).
