# Supplementary material for: What Factors Affect Voluntary Uptake of Community-Based Health Insurance Schemes in Low- and Middle-Income Countries? A Systematic Review and Meta-Analysis
Source: PLoS One. 2016 Aug 31;11(8):e0160479. doi: 10.1371/journal.pone.0160479 (PMC5006971; doi:10.1371/journal.pone.0160479)
Supplement: S3 Table — (DOCX) [file pone.0160479.s003.docx]

| S3 Table: Characteristics of included studies | | | | | | | | | | | | | | | | | | | | | | |
| --- | --- | --- | --- | --- | --- | --- | --- | --- | --- | --- | --- | --- | --- | --- | --- | --- | --- | --- | --- | --- | --- | --- |
| Quantitative | | | | | | | | | | | | | | | | | | | | | | |
| Ref. No  Author (Year)  Study Setting | | Focus of Study | | | Type of Study Design | | | Sample Size(Sampling technique) | | | Method of Analysis | | | Results and Findings | | | | | | | Remarks | |
| 23  Aggarwal A.  (2010)  Yeshasvini, Karnataka, India, (Rural) | | Enrollment:  The study covers various dimensions of vulnerability and assesses their relationship with enrollment and utilization of healthcare in India | | | Cross-Sectional Study | | | 4109-Households ( Multi-stage stratified random) | | | Logit models | | | Education, access to information and SHG membership are empowering factors that increase likelihood of joining and renewing membership. Enrollment disproportionately in favor of wealthier classes although income turns insignificant but has positive relation with probability of enrollment. Poor health status households are more likely to join. Enrollment is positively related with locational vulnerabilities and negatively related with poverty, poor living conditions and distance from government healthcare facilities but shows positive relation towards transport facilities. | | | | | | | Scheme Enrollment is gender neutral at the same time study specified women are major beneficiaries of the program. Enrollment is biased in favour of the empowered classes of the society; | |
| 24  Akotey et al.  (2011)  MHI informal sector, Ghana, (Urban) | Enrollment: The purpose of this paper is to identify the factors which influence the demand for micro-insurance services among the informal sector workers of Ghana who are quite vulnerable to various risks in the economy. | | | | Cross-Sectional Study | | | 100-Individual (Simple Random sampling) | | | Probit Model | | | Premium flexibility, income level and nodal agency are significant determinants of micro-insurance demand. Insurance knowledge, Expectation (trust) and marital status were also found to have positive and significant impact on the demand. | | | | | | | Result also shows that an improvement in the perception of low-income earners about insurers has a positive and significant impact on the demand for micro insurance. Formal education is not a  significant determinant; rather one’s level of insurance knowledge has a positive and significant  Impact on micro-insurance demand. | |
| 27  Bendig et al.  (2011)  Sri Lanka | Enrollment:  Evidence on the determinants of insurance participation using probit models on household survey data from Sri Lanka | | | | Cross-Sectional Study | | | 330-Households | | | Trivariate Probit model Binary Probit models | | | Households with higher assets base are more likely to join. Education of the household head is a strong determinant to join. Household’s experience of a family related shock is positively associated with the participation. Elderly Household heads are less like to join. | | | | | | | Female-headship of a household is positively associated with the enrollment in MFI and the use of micro health insurance whereas household size has negative association. Poorer the Households lower the accessibility to enter in any MFI, resulting less likely to enrol in scheme. | |
| 28  Bhatt R et al.  (2007)  Krupa, Anand, Gujarat, India, (Rural) | Renewal/Drop-out: Factors affecting the decision to purchase health insurance and renewal of insurance in India | | | | Cross-Sectional Study | | | 301-Households | | | Logit model & Heckman two-step method. | | | Customer satisfaction is significant factor in influencing the renewal decision of policyholder. Income is not very significant variable which affect health insurance renewal decision. Education is significant factor which affects renewal decision. | | | | | | | Factors affecting health insurance renewal are not the same as factors affecting health insurance purchase decision. | |
| 29  Bonan et al.  (2011)  Thies, Senegal, (Urban) | Enrollment: Elaborates on various reasons explaining low Enrollment rates in the context of our study in Senegal | | | | RCT | | | 360-Households (Simple Random Sampling) | | | Descriptive and Econometrics analysis is done using probit model | | | Lack of knowledge results in low take-up rates even insurance literacy module has no significant impact on health insurance take-up while marketing treatments have a large and positive significant impact. Study found Enrollment depends more on compensations in the form of reduced fees of membership rather than education. Male-headed household are more likely to join. Both risk aversion and time variable appears not to significant influence Enrollment. | | | | | | | If the state or the city authorities wanted to increase Enrollment rates the most efficient way would be to alleviate the financial barriers to entry.Results indicate that household with recent illness episodes of sickness are not inclined. Enrollment does not depend on whether the head of the household is self-employed or a public servant. | |
| 21  Chankova et al.  (2008)  Ghana Mali and Senegal. Nkoranza, Ghana.  Bla and Sikasso, Mali.  Thies region of Senegal, West Africa, (Both Rural & Urban) | Enrollment:  Investigate the determinants of enrollment impact of MHO membership on use of healthcare services and on OOP healthcare expenditures for outpatient care and hospitalization in Africa (Ghana, Senegal and Mali) | | | | Cross-Sectional Study | | | 2659(Mali), 1806(Ghana) & 1080(Senegal)-Households | | | Multiple logistic regressions and log-linear regression model | | | Lack of information is a cause of Non-enrollment. Study provides the strong evidence, women headed households are more likely to join. Older age of household head is significantly associated with enrollment in Ghana and Senegal. There is a positive relation between employment if the person engaged in agriculture, commerce or administration. Availability of a health facility linked with higher likelihood of enrollment. | | | | | | | A key feature of the Mali and Senegal MHOs benefit packages is that their include outpatient care serviced through primary health facilities. In contrast, the MHO covered in the Ghana study site provides primarily inpatient benefits. | |
| 36  De Allegri, et al. (2006c)  Nouna, Burkina Faso, Ghana, (Both Rural & Urban) | Enrollment:  To identify factors associated with decision to enroll in a community health insurance (CHI) scheme in Burkina Faso, Africa | | | | Case-Control Study | | | 3125-Households | | | Multivariate unconditional logistic regression used to control for possible confounding; Huber-White correction estimates applied to account for potential clustering at community level. | | | Enrollment is associated with Bwaba ethnicity, higher education, highersocioeconomic status, a negative perception of the adequacy of traditional care, a higher proportion of children living within the household, greater distance from the health facility, and a lower level of socioeconomic inequality within the community, but not with household health status or previous household health service utilization. | | | | | | | Individual participatory in another risk-sharing arrangement is not associated with enrollment. No difference between insured and uninsured regarding age and gender. | |
| 37  Donfouet et al. (2012)  Bandjoun, West province of Cameroon, (Rural) | Enrollment:  Spatial interactions in the demand for CBHI in the Cameroon, Africa | | | | Cross-Sectional Study | | | 369-Individuals (Two-stage cluster sampling) | | | To test Bayesian Spatial Tobit Analysis study adopted Gibbs Markov Chain Monte Carlo (MCMC) | | | Solidarity is an important factor of CBHI. Household with more health events are more willing to pay. There is evidence of special interaction as the neighbouring households behave similarly. Higher education positively affect WTP | | | | | | |  | |
| 39  Dong et al. (2005)  Nouna health district, Burkina Faso, (Both Rural & Urban) | Enrollment:  To provide information for devising CBI policies in Nouna Health District of BurkinaFaso. | | | | Cross-Sectional Study | | | 800-Households (Two-stage cluster sampling) | | | Descriptive Statistics with test, Differences in WTP for CBHI analysed using expenditure quintiles; Consumption expenditure collected over a 6-month period | | | Chances of enrollment poor people are low if premium is not adjusted for Income or no exemptions or subsidies are provided. | | | | | | | Gini coefficient of household WTP twice of individual WTP suggesting a reason for household enrollment being better than individual. | |
| 40  Dong et al. (2009)  Nouna, Burkina Faso, (Both Rural & Urban) | Renewal/Drop-out:  The study explores the reasons for members who are not willing to renew their membership. | | | | Cross-Sectional Study | | | 1309-Households (Two-stage cluster sampling) | | | Logistic regression, Chi-square tests used to assess differences in proportions. | | | Affordability, Female headed household, higher age or lower education of a household head, lower number of illness episodes in the past three months, fewer children or elderly in a household, poor perceived healthcare quality, less seeking care in the past month and living in rural area have positively affect drop-out. Higher household expenditure and a shorter distance to the contracted health facility increase the drop-out. | | | | | | | High drop-out rates endanger the sustainability of CBI not only because they reduce the size of the insurance pool, but also because they bear a negative impact on further enrollment and drop-out. | |
| 41  Dror DM. (2010)  Maharashtra, Karnataka and Bihar, India, (Rural) | Enrollment:  Examination of the association between insurance status and indicators on social-capital in states of Maharashtra, Karnataka and Bihar in India. | | | | Case-Control Study | | | 700-Households (Two-staged sampling) | | | Descriptive with nonparametric statistical analysis. | | | Trust seems to be a vital (yet insufficient) precondition for success in achieving voluntary affiliation, Finance emerged as the second most important reason for not joining in 3 out of 4 locations. Among the non-insured cohort, the main reason for not joining was lack of trust on the part of scheme-provider. Access to quality care mentioned as the positive factor of joining the scheme. | | | | | | | Interactions of the community with a solidarity promoting organization (such as an MIU), even when it comes from the outside, can enhance trust and social capital. | |
| 42  Eckhardtet al.  (2011)  El Páramo, Ecuador., (Rural) | Enrollment:  To assess the willingness to pay and its factors in CBHI in El Paramo, Ecuador. | | | | Cross-Sectional Study | | | 210-Household (Two-stage cluster sampling) | | | Descriptive with nonparametic statistical analysis to test significance | | | Willingness to join was found to be negatively associated with education. Enrollments are likely to be lower than the stated willingness to join, still CHI scheme presents as an interesting financing alternative in rural areas | | | | | | | With affiliation, 92.2percent of interviewees stated that they would visit the local health facility more often. This clarifies that people who have clear foresight of getting ill are more willing to join the scheme. | |
| 43  Fonta et al. (2010)  Enugu State, South-eastern Nigeria, (Rural) | Enrollment:  Paper examines the possibility of adopting CBHI using in-kind payments in rural Nigeria. | | | | Cross-Sectional Study | | | 380-Households (Simple Random Sampling) | | | Contingent-Valuation method used & Estimation done through probit | | | Household members who have foresight of getting sick are more likely to join. Distance (high cost of transportation), Education and available quality of health services have positively related with enrollment. | | | | | | | Household heads that have greater trust and confidence in the proposed scheme are willing to pay higher amounts to enroll than those who have low confidence in the scheme. | |
| 44  Gnawali et al.  (2009)  Nouna, Burkina Faso, Ghana, (Rural) | Enrollment:  To quantify the impact of CBI on utilisation of health-care services in Burkina Faso. | | | | Cross-Sectional Study | | | 990-Households (Cluster Random Sapling) | | | Propensity Score Matching estimated by logistic regression. | | | Per-capita expenditure in richest quartile, household size, household with more children below 5years of age, education of household heads and involvement in any other risk sharing network is positively linked with Enrollment. Younger household heads are less likely to enrol whereas premium subsidies have the positive relation. | | | | | | | The policy implications suggested are (a) there is a need to subsidize the premium to favour the enrollment of the very poor(b) Various measures need to be in place in order to maximize the population’s capacity to enjoy the benefits of insurance once insured. Though HH perceived good quality of care however did not enroll. Possibly due to unaffordability. | |
| 45  Gumber A. (2001)  SEWA, Ahmedabad, Gujarat, (Both Rural & Urban) | Enrollment:  Paper examines the determinants of enrollment in CBHI using household data from pilot study undertaken in Gujrat, India. | | | | Cross-Sectional Study | | | 1200-Households (Purposive sampling ) | | | Multinomial Logit model. | | | Information availability (scheme) is positively related with enrollment. Rate is higher for women with chronic illness or who has an incidence of illness in last 1year. Enrollment is neutral amongst different quintiles. Enrollment rate declines with increasing household size. Education is positive related with enrollment. There is urban bias in enrollment. | | | | | | | Hospitalisation Coverage is most preferred by rural and urban population. The community plan fairly addresses equity in enrollment but that, in terms of providing financial protection, social insurance coverage is much more successful. | |
| 46  Ito et al. (2009)  Yeshaswini, Karnataka, India, (Rural) | Enrollment:  Investigation of insurance Enrollment decision in Yeshashwini Scheme, operating in Karnataka, India. | | | | Cross-Sectional Study | | | 209-Households (Purposive random sampling) | | | Probit Analysis based on Expected Utility theory and Prospect Theory. | | | Households with healthy head members are more likely to be enrolled. This is due to the fact that ill member if head, would have less money to spare. Household with sick head member would have low income and hence is unlikely to enroll. Evidence of existence of adverse selection is seen. | | | | | | | We find some evidence that people behave risk-lovingly when facing risk of losses, which is consistent with prospect theory and insurance covers losses. We also find that hyperbolic discounters are more likely to purchase the insurance, which can be explained by demand for commitment, which sophisticated hyperbolic discounters have. | |
| 47  Jutting J.P. (2003)  les mutuelles de santés, Senegal, (Rural) | Enrollment:  Study deals with the subject of participation in local development organisations and institutions in rural areas of Senegal. | | | | Cross-Sectional Study | | | 360-Households (Two-stage sampling) | | | Binary Probit Model was used | | | Income is significantly and positively related with participation. Governance on management and finances was negatively related to membership in Sanghe mutual.Household heads with previous experience of membership in local organisation tend to participate more. Types of health insurance provided (Primary healthcare in Ngaye Ngaye and in-patient care in others) have not affected decision to participate significantly. | | | | | | | Household head of the family seems to be better educated. Poorest of the poor within the villages find it financially difficult to participate. Being a Christian increases the probability of being a member by roughly 37percent. People from Wolof ethnicity have a high disposable income and more likely to be a member as compared to Serere and Peulh. | |
| 49  Kuwawenaruwa et al. (2011)  Tiba Kwa Kadi (CHF/ TIKA); (Morogoro, ilala, and Kinondoni), (Kigoma, Kilosa, Mbulu and singida), Tanzania, (Urban) | Enrollment:  To assess the willingness to pay of people and their response to change in Benefit Package and Scheme Design in CHF scheme in three urban councils; Kigoma, Kilosa, Singida in Tanzania. | | | | Cross-Sectional Study | | | 2724-Individual | | | Logit Model, Bi-variate analysis was done for Willingness to Pay and Willingness to Join, Statistical significance analysed through Pearson chi-square and the Mann-Whitney U test used for estimation of WTP and WTJ. | | | Households in Dar es Salaam who possess higher income, are educated and having better access to healthcare are more likely to pay and join.People with formal education and employment will be more willing to pay and join. Fixed premium for household entails enrollment of bigger households are more.Poor people whose self-assessed health is poor will be more willing to join than healthy ones.WTP for insurance is likely to reduce due to lower income levels of Age. | | | | | | | Insured are more likely to get married. Those who are eligible for exemptions were less likely to join. | |
| 50  Lammers et al. (2010)  Lagos, Nigeria, (Urban) | Enrollment:  Study about the determinants to join recently launched low-cost health insurance scheme in Nigeria. | | | | Cross-Sectional Study | | | 677-Households, 1941-individual | | | Logit estimation used. | | | Low wealth, small household size, high-risk preference, health optimism, and underestimation of health risks explain a lower Enrollment propensity. Households with higher product awareness are more likely to be enrolled. Health risk occurrence and lower self-assessed health increases the propensity to be enrolled significantly (This strongly suggesting adverse selection). Ethnicity and religion appear to be important determinants in the insurance decision as basic model shows that Muslims have higher propensity to enrol than other ethnicities. | | | | | | | The propensity to enroll is seven times higher for persons from highest quintiles; however WTP of wealthy household does not mean a necessary enrollment as health shocks are less frequent in wealthy household. The elderly aged (>49) do not have larger propensity to enroll through they have higher need for healthcare. | |
| 51  Liu et al. (2013)  NCMS, China, (Rural) | Enrollment:  Examines the role of social learning in household enrollment decision for the New Cooperative Medical Scheme in rural China. | | | | Cohort Study | | | 3266-Households (Multi-stage Random cluster-sampling) | | | Panel data analysis is done using fixed and random efect models of 3 waves of longitudinal nation-wide survey employed for model estimates, to control for the endogeneity of the village-level peer enrollment level. | | | Low household income and community urbanicity indicators resulted significant negative coefficients.Study highlighted an interesting finding of 10-percentage-point increase in the enrollment rate in a village increases one’s take-up probability by 5 percentage points (social multiplier effect of 1.9 at the village level). | | | | | | | Wealthier and relatively well-educated older male household heads with Han nationality tend to be opinion leaders in NCMS enrollment. | |
| 22  Mathiyazhagan K. (1998)  Karnataka, India, (Rural) | Enrollment:  Examining Willing ness to Pay and policy concerns for CBHI in Karnataka in India. | | | | Cross-Sectional Study | | | 1000-Households (Multi-stage sampling) | | | Logistics Model, Contingent-Valuation Method to elicit WTP | | | Income is significantly and positively related with participation. HH size positively influenced the decision making for willingness to join and pay. Large households had 119percent higher probability to join and 27percent higher chance to pay. Longer illness experience, education and distance have positive and significant contribution in joining whereas Age and Caste is inversely related to WTJ. | | | | | | | Probability of willingness to pay for a rural health insurance scheme was found to be less than the probability of willingness to join (WTJ). | |
| 52  Mladovsky P. (2014)  Senegal, (Rural) | Renewal/Drop-out: The study explores whether never having actively participated in CBHI is a determinant of dropout in Senegal. | | | | Case-Control Study | | | 382-Households | | | Logit model was used to assess the probability to retain the membership | | | Most of renewed households are wealthier and have higher expenditure than those who dropped-out (although not significant). Satisfaction with the accessibility of premium price was quite low and not significant. Odds ratios of retaining in the scheme for demographic, education, ethnicity and religion variables are also not significant, except for age. Households who have foresight of illness, accident, injury or disability, easy and quality access to health service, source of information and knowledge are significantly positive relation with retaining in scheme. | | | | | | | Training is the most highly correlated with renewals, followed by voting, participating in a general assembly, awareness raising / information dissemination and informal discussions / spontaneously helping. Perceived trust worthiness of the scheme management / president; accountability and being informed of mechanisms of controlling abuse/fraud are also significantly positively correlated with remaining in the scheme. Perception of poor quality of health services is identified as another most important determinant of drop-out; Financial factors do not seem to determine drop-out. | |
| 54  Msuya et al.  (2004)  Igunga, Tanzania, (Rural) | Enrollment:  To evaluate the role of the community health funds in lowering the barriers to access healthcare in Tanzania | | | | Case-Control Study | | | 100-Households (Multi-stage sampling) | | | Probit analysis | | | Village of residence, Ethnic origin,Main occupation of the household head, Education level of the key female member, household size and the wealth status of the household have statistically significant relationship with CHF status whereas ethnicity, gender and education of household head shown insufficient coefficient. | | | | | | | Income is most important factor determining household participation.This result showed that even though community insurance schemes were advocated as one important means to reach the poorest of the poor, it has not happened in the case of the Igunga CHF scheme. | |
| 55  Msuya et al.  (2007)  Igunga, Tanzania, (Rural) | Enrollment:  This study aims to evaluate the role of the community-health funds (CHF) in lowering the barriers to assessing healthcare in Tanzania | | | | Cross-Sectional Study | | | 200-Households, 1700-Individual (Multi-stage sampling) | | | Probit analysis | | | 1percent point increase in income was likely to increase the probability of joining the scheme by 12.5percent. Households with big family size are more likely to join as the premium is independent of family size. Education variable was found to be insignificant. Igurubi (near to health facility) showed higher propensity to enroll than Itumba (further village). | | | | | | | Members of a community health fund are more likely to seek formal medical care when they are ill than non-members. | |
| 56  Noubiap et al. (2013)  Bonassama, Doula, Cameroon, (Rural) | Enrollment:  To evaluate CBHI knowledge, concern and preferences of informal sector workers in Bonassama Health District of Doula, Cameroon. | | | | Cross-Sectional Study | | | 160-Individual (Simple Randon Sampling) | | | Descriptive, Chi-square test or its equivalents were used to compare qualitative variables | | | Profession, ethnicity, access to healthcare and religious affiliation of its members are directly linked with enrollment. Whereas lack of awareness is inversely related to employment. | | | | | | | Lack of awareness and limited knowledge on the basic concepts of a CBHI by this target population as one of the reason for low enrollment. Solidarity based community associations to which the vast majority of this target population belong are prime areas for sensitization on CBHI schemes. | |
| 57  Nsiah-Boateng, et al.  (2013)  Ga District, Greater Accra, Ghana, (Urban) | Enrollment:  The study assessed the performance of Ga District Mutual Health Insurance Scheme, Greater Accra region, Ghana | | | | Case-Control Study | | | 376-Individual (Multi-Stage sampling) | | | Descriptive without test | | | Study has reported reasons for not enrolling are expensive contribution and Scheme does not offer services needed.Lack of education and insurance knowledge leads to lesser enrollment. | | | | | | | Increasing trends in membership coverage and revenue are largely driven by the exempt groups and subsidies from the NHIA. | |
| 58  Onwujekwe et al. (2009)  Igboukwu and Neni communities in Anambra, Nigeria, (Both Rural & Urban) | Enrollment:  To determine how equitable enrollment and utilisation of CBHI is for two communities Igboukwu and Neni in Anambra State of Nigera | | | | Cross-Sectional Study | | | 455(Igboukwu), 516(Neni)-Household (Simple Randon Sampling) | | | Descriptive with test, Principal components analysis | | | Level of awareness of both schemes which is important for enrollment is high. Unavailability of doctors was reported by most of the respondents; hence it should be sorted to increase enrollment. Cost of registration is a hindrance. There is a demand for scheme awareness so that it is successful. | | | | | | | Enrollment is generally low and contributions are retrogressive. The average premiums also small. Major reason unwillingness because of 1. Cost of registration is high 2. Unavailability of doctors There is need for increase in pool of funds, risks and subsidies from government and donors in order to ensure equitable financial risk protection. | |
| 59  Onwujekwe et al. (2011)  Enugu and Anambra, Southeast Nigeria, (Both Rural & Urban) | Enrollment:  Information about the determinants in Enugu and Anambra States, Nigeria | | | | Cross-Sectional Study | | | 3070-Household (Simple Randon Sampling) | | | Logistic regression with principal components analysis and Contingent valuation method | | | WTP positively related to health expenditure using OOP expenses. WTP is positively related to SES and education. Household size is negative related to joining. Geographical area of residence is not a barrier to join under the scheme. | | | | | | | There were high levels of catastrophic costs, but with appreciable levels of affordability and altruistic WTP for CBHI, coverage can be increased and financial risk protection assured for most people that need CBHI. | |
| 61  Oriakhi et al.  (2012)  Edo state, Nigeria, (Rural) | Enrollment:  To find out the factors which influence the willingness to participate in rural areas at Edo State, Nigeria | | | | Cross-Sectional Study | | | 360-Household (Multi-stage random sampling) | | | Logistics regression multi-stage sampling procedure | | | Household size and Membership of formal organization have significantly positive relation with participate. Education is negatively related with enrollment. Nature of employment and Income (significantly) negatively related with willingness to participate whereas medical expense and credit obtained for medical treatment both are positively related with participate. Low trust in the management leads to low enrollment rate. | | | | | | | The study recommended the incorporation of community participation in the scheme especially in scheme management selection and large household have an encouragement to participate in CBHI while awareness creation as measures to promote CBHI scheme in the state. | |
| 63  Panda et al. (2013)  Uttar Pradesh and Bihar, India, (Rural) | Enrollment:  Study examines what drives the Enrollment, the degree of inclusive practices of the schemes and influence of health status on enrollment in rural Uttar Pradesh and Bihar, India. | | | | Cross-Sectional Study | | | 369(Bihar), 1711(Uttar Pradesh)-Households (Cluster sampling) | | | Marginal-effect estimates based on Logit specification. | | | Household’s socio-economic status does not appear to substantially inhibit Enrollment. In some cases scheduled caste/scheduled tribe households are more likely to enroll. Households with greater financial liabilities find insurance more attractive. Access to the national hospital insurance scheme Rashtriya Swasthya Bima Yojana does not dampen CBHI Enrollment. Households with children seem to be more risk averse and expect a higher need for health case so are more likely to enroll. | | | | | | | Coverage of transportation cost in benefit-package works towards reducing the potential negative effect of accessibility on Enrollment. Education affects positively the up-take however it is restricted to Vaishali. Intra-household pooling of income as a measure of risk pooling can help smooth consumption and exerts a negative effect on Enrollment of insurance. None of the locations show that low castes communities are less likely to enroll. | |
| 65  Ranson M K  (2001)  Armenia, (Rural) | Enrollment:  Assesses the impact of the Self-Employed Women’s Association’s (SEWA’s) Medical Insurance Fund, Gujarat, in terms of inclusion of the poor, hospital utilization, and expenditure. | | | | Cohort Study | | | 700-Households (Two-stage random cluster sampling) | | | logit model and log-linear model | | | Older age and higher frequency of illness episode in the last month are significantly associated with membership. Quintile of ESI (Economic Status Index) is taken as proxy of wealth is not statistically associated with membership in the fund. Fund members have higher rates of hospitalization (even women living in the same households as fund members) but this association was not significant. | | | | | | | Women of age 30 years and above were 3.4 times as likely to join the fund as those of 18 to 20 years. Each additional illness reported within the last month (acute illnesses as well as exacerbations of chronic disease) was associated with a 70 percent to 80 percent (best fit) increase in the probability of joining the Fund. Lack of awareness of benefits among fund members or costs and difficulties associated with submitting an insurance claim. | |
| 66  Rao K. et al.  (2009)  Afghanistan, (Rural) | Enrollment:  Performance of one type CBHI scheme, the community health fund, which was piloted for the first time in five provinces of Afghanistan. | | | | Cross-Sectional Study | | | 160(Parwan), 160(Saripul)-Households | | | Descriptive Statistics with test, Quasi-experimental design (one-group pretest-posttest design); Control of spill-over effect as no other programme operated in the catchment area during the study period. | | | Unawareness on part of scheme; high premiums; and perceived low quality of services at the CHF clinics are the main reason among non-member for not enrolling. Low perceived service quality affects enrollment: specifically lack of trust in doctor’s skills and lack of drugs. | | | | | | | Poorest and female-headed households were enrolled into the programme free of cost. Further poor households are eligible for reduced premiums | |
| 68  Schneider et al. (2001)  Byumba, Kabgayi and Kabutare, Rwanda, (Rural) | Enrollment:  Whether health insurance membership improve financial accessibility to care without increasing the burden of OOP health expenditure in three districts of Rwanda | | | | Cross-Sectional Study | | | 2518-Households, 11582-Individual | | | Logit regression | | | Literate household head 103percent more likely to enroll than illiterate. Large households (4+ members) are 60percent more likely to buy insurance than smaller households. Households who live within 30 minutes of their health facility have a 296percent higher probability of joining than those who live farther away. | | | | | | | Households who own a radio (awareness campaign) is 47percent more likely to enroll Male-headed households are 55percent more likely to join than female-headed and households with pregnant women are 23percent more likely to join, although these results are not significant. Cattle ownership and different income quartiles were not significant in the demand for health insurance. | |
| 69  Shafie et al.  (2013)  Penang Malaysia, (Urban) | Enrollment:  To assess the willingness of Malaysians to participate in a VCHI plan of Malaysia | | | | Cross-Sectional Study | | | 472-Individuals (Two-stage cluster sampling) | | | Multinomial logit regression model | | | Married individuals are almost three times more likely to choose VCHI. Chinese as a group are more risk averse and so have a higher WTP. The Enrollment is positively affected by higher income. Enrollment is positive related to education. | | | | | | | Contribution payable is influenced by ethnicity, educational level, household monthly income, the presence of chronic disease and the presence of private insurance coverage | |
| 73  Wang H. et al.  (2005) Fengshan Township, Guizhou, China, (Rural) | Enrollment:  Study evaluates the probability of farmers joining a re-established CBI Fengshan Township, Guizhou Province in China. | | | | Cross-Sectional Study | | | 1173-Households, 4160-Individuals (Multi-stage sampling) | | | Logistic regression | | | Income is an important factor influencing farmers’ decision to join a CBI despite the premium representing a very small fraction of household income.  Farmers self-perceived good health are less likely to participate in the CBI than farmers with medium or poor health status; these results are statistically significant comparing good with poor health status. | | | | | | | Income and health status influence enrollees’ utilization of health services: richer/sicker participants, meaning that poorer/healthier participants subsidize the rich/sick. Wealthy farmers benefit the most from the CBI with low premium and high co-payment features. In conclusion, policy recommendations related to the improvement of the benefit distribution of CBI. | |
| 74  Zhang L et al.  (2006)  Fengsan Township, Guizhou, China , (Rural) | Enrollment:  Examine the probability of farmers’ willingness-to-join with emphasis on social capital in China | | | | Cross-Sectional Study | | | 1157-Households, 2830-Individuals (Multistage sampling) | | | Logistic regression model with odds ratios (ORs) estimation in this study, Discrete choice model to predict WTJ | | | Analysis showed both individual level trust index and community level reciprocity index are significantly and positively associated with the probability of farmers’ WTJ the CHI. Financial social support and the probability of WTJ the CHI showed positively and significantly association. Both income and asset has strongly positive associations with the probabilities of WTJ. Age, Medical expense and farmers who reside closer to village health facility are more willing to join whereas distant residents are less likely to join. | | | | | | | The results imply that the participation rate of CHI might be increased by enhancing social capital in rural China. However, social capital is affected by many socio-economic factors, such as income inequality. | |
| Qualitative Studies | | | | | | | | | | | | | | | | | | | | | | |
| Ref No. Author  (Year)  Study Setting | | | Participants | | | | Sampling and Data Collection (Response) | | | Focus of Paper | | | | | Themes covered | | | | Method(Analysis) | | | |
| 75  Atim et al. (2000)  Nkoranza Community Financing Health Insurance Scheme, Ghana (rural) | | | Subscribers | | | | A Stratified multi-stage samplingFGDs (43), Interviews with 300 individuals | | | An external evaluation of the Nkoranza Community Financing Health Insurance Scheme, Ghana | | | | | KQTBA | | | | Cross-Sectional Studies(Descriptive Statistics) | | | |
| 30  Basaza et al. (2007)  Ugandan Community Health Insurance Scheme, Uganda (rural) | | | Scheme members | | | | KI interviews (23), EI interviews (39), Total (62) | | | To explore the reasons for the limited success of CHI | | | | | KTBRAL | | | | Case Study Research Design (Framework method) | | | |
| 31  Basaza et al. (2008)  Ugandan Community Health Insurance Scheme, Uganda (rural) | | | Members and non-members | | | | FGDs (30), Interviews (18) | | | To study the reasons for low enrollment in two different models of CHI | | | | | KQTBRAD | | | | Cross-Sectional Studies Analysis (NR) | | | |
| 32  Basaza  (2010)  Ugandan Community Health Insurance Scheme, Uganda (rural) | | | District Health Officers and senior staff of the Ministry of Health | | | | Purposive sampling Interviews (32) | | | To investigate the knowledge of CHI and the perception of its relevance by key policy makers and health service managers | | | | | KL | | | | Cohort Studies(Framework method) | | | |
| 33  Criel et al.  (1998)  CBHI in Bwamanda, Democratic Republic of Congo (rural and urban ) | | | Subscribers and non-subscribers | | | | FGDs (10) | | | To find pertain to the reasons for people to subscribe to the scheme | | | | | KQTBA | | | | Cross-case analysis | | | |
| 76  Criel et al.  (2003)  CBHI in Bandjoun, West province of Cameroon (rural) | | | Subscribers and non -subscribers | | | | FGDs (12) | | | To study the reasons for drop out from the CBHI scheme | | | | | | KQTBA | | | | Cross-Sectional Studies Cross analysis | | |
| 34  De Allegri et al.  (2006_a_)  Nouna Health District, Burkina Faso, Ghana (rural and urban) | | | Household heads | | | | Stratified Purposive Sampling Interviews (32)(Male heads, 24 and Female head, 8) | | | To assess determinants of enrollment in a newly established CBI scheme | | | | | | KQTBCAD | | | | Cross-Sectional Studies, Grounded theory (Contrast and compare method | | |
| 35  De Allegri et al.  (2006_b_)  Nouna Health District, Burkina Faso, Ghana (rural and urban) | | | Insured and non-insured members | | | | Stratified Purposive Sampling Interviews (32),10 FGDs | | | To provide adequate policy guidance to decision makers in LMIC by producing an in-depth understanding of how consumers’ preferences may affect decision to participate in such schemes | | | | | | KQTBA | | | | Cross-Sectional Studies, (Method of Constant Comparison) | | |
| 48  Kyomugisha et al.  (2009)  community health insurance schemes (CHI) in Uganda (rural) | | | Members and non-members and KI are Scheme managers, officials from Ministry of health and one health financing organisation | | | | Purposive sampling FGDs (15), KI (12) | | | To examines issues of equity and sustainability in CHI schemes, which are prerequisite to health sector financing | | | | | | KQTBRL | | | | Cross-Sectional Studies Analysis(Descriptive) | | |
| 64  Poletti et al. (2007)  Rural Setting, community health insurance schemes (CHI) in Uganda (rural) | | | High level government officials, heads of hospitals and polyclinic, family physician, major donors, academicians and consultants, NGO, Health post nurses, NGO partner, heads of village council, | | | | Snowballing technique FGDs (02) and 30 (KI) | | | To identify the major constraints and opportunities for scaling up community-based health insurance in Armenia | | | | | | KQTBADL | | | | Case Series and Case Reports, (Grounded theory approach) | | |
| 67  Schneider P. (2005)  Community-Based Health Insurance in Rwanda (rural) | | | MHI members, Non-members, MHI managers, Healthcare provides | | | | FGDs (24) | | | To identify trust-building factors in the provider-consumer-MHI relationship that motivate consumers to insure | | | | | | KQTL | | | | Case-Control, Exploratory Study (descriptive nalysis) | | |
| 71  Turcotte-Tremblay AM et al.  (2012)  Mutual health organizations (MHO) in Benin, Senegal (rural and urban) | | | MHO promoters, Technicians, Elected members, Health professional | | | | Snowball approach FGDs (02), Interviews (23) (10 promoters representatives, two coordinators, one technical assistant, eight elected members, one healthcare member, one healthcare manager, one medical doctor, eight elected members and six healthcare members | | | To map initiative implemented to increase the pool of MHO members in Benin | | | | | | KQTBDL | | | | Multiple Case study design, (content analysis) | | |
| 6 Mixed-method papers | | | | | | | | | | | | | | | | | | | | | | |
| S.No.  Ref. No  Author  (Year)  Study Setting | | | | Focus of Study | | Type of Study Design | | | Sample Size(Sampling technique) | | | Method of Analysis | | | | | | Results and Findings | | | | Remarks |
| 25  Alatinga et al. (2011)  Kassena-Nankana East Scheme, Ghana, (Rural) | | | | Enrollment:  The impact of Mutual Health Insurance on access and quality of healthcare for the rural poor in Northern Ghana | | Cohort Study | | | 100-Individual (cluster random sampling ) | | | Descriptive Statistics with test, Cramer’s V correlation coefficient | | | | | | Positive relationship of insurance and insurance status. Whereas distance to the health facility prevents households from enrolling in MHIS. Flat rate nature of insurance premium is preventing majority of households from enrolling in health schemes | | | | Insured are generally the rural middle class with relatively higher level of incomes. |
| 38  Dong et al. (2004)  Nouna, Burkina Faso, (Both Rural & Urban) | | | | Enrollment:  Studies the acceptability and sustainability of the CBI scheme in Nouna health district of Burkina Faso through eliciting Willingness to Pay for the scheme. | | Cross-Sectional Study | | | 160-Households (Purposive sampling) | | | Logistic regression analysis | | | | | | Mean and median WTP increased with household size and proportion of children. Young males preferred to pay more than the elder ones. Preference was to cover drug, lab tests, impatient stags and surgery in the package. Community participation and solidarity necessary for CBHI success. Marketing found important variable in initial designing of the scheme to keep the membership high. | | | | The average household premium for the insurance based on the median household head’s WTP is about 6.3percent of the annual household expenditure. However, it is needed to have more support for the success of the CBI. |
| 62  Ozawa et al. (2009)  Cambodia, (Rural) | | | | Enrollment:  To understand the role and influence of villager’s trust for the health insurer on enrollment in a CBHI scheme in Cambodia | | Cohort Study | | | 560-Hoseholds (Stratified random sampling) | | | Multinomial logistic regression models Multivariate regression models | | | | | Significant association is found between insurer trust levels and CBHI enrollment. Trust factor of renewed members are significantly more than those who are new to the scheme or drop outs. Other factors affordability of premium time of premium collection and understanding of insurance showed positive relation with enrollment however Income does not play significant role. | | | | | Five domains of insurer trust were identified: organizational trust, financial trust, honesty, competence, and personal interactions. Individual who was never insured tended to have more years of education. |
| 70  Sinha et al. (2006)  VIMO SEWA, Ahmedabad, Gujarat, (Urban) | | | | Renewal/Drop-out:  Interest of the paper is to find out the how VIMO SEWA can protect its members and increase membership in India | | Cohort Study | | | 220(Purposive random sampling) | | | Descriptive with test | | | | | Most important factor for not renewing membership is not being approached by a Vimo SEWA aagewan (grassroots worker). Lack of money and Individual characteristics like age, education, and occupation to buy insurance do not appear to be major factors affecting the member’s renewal decision but time of collection and scheme understating considerably affect. | | | | | A high renewal rate also contributes to the financial viability and efficiency of the scheme. Members who have been in the scheme for a length of time develop a sound understanding of the scheme. |
| 58  Onwujekwe et al. (2010)  CBHI in Enugu and Anambra, Southeast Nigeria (rural and urban) | | | | Scheme members | | FGDs (12), Interviews (3070) | | | To examine socio-economic status (SES) and geographic differences in willingness of respondents to pay | | | | B | | | | Cross-Sectional Studies, Mixed Methods (Content analysis) | | | | |  |
| 72  Uzochukwu et al. (2009) Community Based Health Insurance Scheme in Anambra State, Nigeria (rural) | | | | Policy makers and managers and CBHI members and health workers | | FGDs (08), Interviews (14) (1 senior politician, 8 state policy makers and 5 LGA officials), Health workers (4), Managers of the scheme (2), CBHI and Non-CBHI members (8 FGDs), Members of the community health committees (16) | | | To explore the CBHI policy development and implementation process and the factors that have constrained or enhance its implementations | | | | KQTBRAL | | | | Case study Mixed Methods, (Principal Components Analysis (PCA)) | | | | |  |
