# Supplementary material for: What Factors Affect Voluntary Uptake of Community-Based Health Insurance Schemes in Low- and Middle-Income Countries? A Systematic Review and Meta-Analysis
Source: PLoS One. 2016 Aug 31;11(8):e0160479. doi: 10.1371/journal.pone.0160479 (PMC5006971; doi:10.1371/journal.pone.0160479)
Supplement: S1 Text — (DOCX) [file pone.0160479.s004.docx]

S1 Text: Search Strategy

Ovid MEDLINE(R), Ovid MEDLINE(R) In-Process & Other Non-Indexed Citations, Ovid MEDLINE(R) Daily and Ovid OLDMEDLINE(R) 1946 to Present

18th Nov 2013

1. Developing Countries.sh,kf.

2. exp Africa/ or exp Asia/ or exp Caribbean/ or exp West Indies/ or exp South America/ or exp Latin America/ or exp Central America/

3. (Africa or Asia or Caribbean or West Indies or South America or Latin America or Central America).tw.

4. exp Russia/ or (Afghanistan or Albania or Algeria or Angola or Antigua or Barbuda or Argentina or Armenia or Armenian or Azerbaijan or Bangladesh or Barbados or Benin or Byelarus or Byelorussian or Belarus or Belorussian or Belorussia or Belize or Bhutan or Bolivia or Bosnia or Herzegovina or Hercegovina or Botswana or Brazil or Bulgaria or Burkina Faso or Burkina Fasso or Upper Volta or Burundi or Urundi or Cambodia or Khmer Republic or Kampuchea or Cameroon or Cameroons or Cameron or Camerons or Cape Verde or Central African Republic or Chad or Chile or China or Colombia or Comoros or Comoro Islands or Comores or Mayotte or Congo or Zaire or Costa Rica or Cote d'Ivoire or Ivory Coast or Croatia or Cuba or Djibouti or French Somaliland or Dominica or Dominican Republic or East Timor or East Timur or Timor Leste or Ecuador or Egypt or United Arab Republic or El Salvador or Eritrea or Ethiopia or Fiji or Gabon or Gabonese Republic or Gambia or Gaza or Georgia Republic or Georgian Republic or Ghana or Gold Coast or Grenada or Guatemala or Guinea or Guam or Guiana or Guyana or Haiti or Honduras or India or Maldives or Indonesia or Iran or Iraq or Jamaica or Jordan or Kazakhstan or Kazakh or Kenya or Kiribati or Korea or Kosovo or Kyrgyzstan or Kirghizia or Kyrgyz Republic or Kirghiz or Kirgizstan or Lao PDR or Laos or Latvia or Lebanon or Lesotho or Basutoland or Liberia or Libya or Lithuania or Macedonia or Madagascar or Malagasy Republic or Malaysia or Malaya or Malay or Sabah or Sarawak or Malawi or Nyasaland or Mali or Marshall Islands or Mauritania or Mauritius or Agalega Islands or Mexico or Micronesia or Middle East or Moldova or Moldovia or Moldovian or Mongolia or Montenegro or Morocco or Ifni or Mozambique or Myanmar or Myanma or Burma or Namibia or Nepal or Netherlands Antilles or New Caledonia or Nicaragua or Niger or Nigeria or Northern Mariana Islands or Oman or Muscat or Pakistan or Palau or Palestine or Panama or Paraguay or Peru or Philippines or Philipines or Phillipines or Phillippines or Papua New Guinea or Portugal or Romania or Rumania or Roumania or Russia or Russian or Rwanda or Ruanda or Saint Lucia or St Lucia or Saint Vincent or St Vincent or Grenadines or Samoa or Samoan Islands or Navigator Island or Navigator Islands or Sao Tome or Senegal or Serbia or Montenegro or Seychelles or Sierra Leone or Sri Lanka or Ceylon or Solomon Islands or Somalia or Sudan or Suriname or Surinam or Swaziland or South Africa or Syria or Tajikistan or Tadzhikistan or Tadjikistan or Tadzhik or Tanzania or Thailand or Togo or Togolese Republic or Tonga or Trinidad or Tobago or Tunisia or Turkey or Turkmenistan or Turkmen or Uganda or Ukraine or Uruguay or USSR or Soviet Union or Union of Soviet Socialist Republics or Uzbekistan or Uzbek or Vanuatu or New Hebrides or Venezuela or Vietnam or Viet Nam or West Bank or Yemen or Yugoslavia or Zambia or Zimbabwe).tw.

5. ((developing or less* developed or under developed or underdeveloped or middle income or low* income or underserved or underserved or deprived or poor*) adj (countr* or nation? or population? or world or state*)).ti,ab.

6. ((developing or less* developed or under developed or underdeveloped or middle income or low* income) adj (economy or economies)).ti,ab.

7. (low* adj (gdp or gnp or gross domestic or gross national)).tw.

8. (low adj3 middle adj3 countr*).tw.

9. (lmic or lmics or third world or lami countr*).tw.

10. transitional countr*.tw.

11. or/1-10

12. insurance, health/ or insurance, major medical/ or managed care programs/ or not-for-profit insurance plans/ or prepaid health plans/ or Insurance Coverage/ or Universal Coverage/

13. ((health or health-care or healthcare or medical) adj3 (insurance or microinsurance or micro-insurance)).ti,ab.

14. ((prepaid or pre-paid or "not for profit" or not-for-profit) adj3 plan*).ti,ab.

15. 12 or 13 or 14

16. (enroll* or adopt* or uptak* or uptake or willingness-to-pay or "willingness to pay" or willing-to-pay or "willing to pay" or uptake or choos* or support or demand* or voluntary or community-based).ti,ab.

17. Choice Behavior/ or Patient Preference/

18. 16 or 17

19. 11 and 15 and 18

20. limit 19 to yr="1990 -Current"

21. exp Animals/

22. Humans/

23. 21 not (21 and 22)

24. 19 not 23 [Narrow Search 1,633 hits]

25. 11 and 15

26. limit 25 to yr="1990 -Current"

27. 26 not 23 [Broad Search – 6,524 hits]

## 
