# Supplementary material for: What Factors Affect Voluntary Uptake of Community-Based Health Insurance Schemes in Low- and Middle-Income Countries? A Systematic Review and Meta-Analysis
Source: PLoS One. 2016 Aug 31;11(8):e0160479. doi: 10.1371/journal.pone.0160479 (PMC5006971; doi:10.1371/journal.pone.0160479)
Supplement: S2 Text — (DOCX) [file pone.0160479.s005.docx]

## S2 Text: Data Extraction Sheet

1. Total citations imported (15,770)
2. Duplicates removed (4,372)
3. First screening: title and abstract screening (11, 398)

- Exclude on date
  *Exclude studies prior to 1990*
- Exclude on country
  *Exclude if study is not carried out in a low or middle income country*
- Exclude on topic
  *Exclude if study is on other health insurance mechanisms (private and social) or other topics like microfinance*
- Include based on title and abstract
  *Cannot be excluded so is marked as included. These studies will further require full report retrieval.*
- General exclusion
  *Exclude studies if found completely irrelevant.*
- Overlapping
  *Exclude unidentified duplicates*

1. Second screening: screen on full report (905)

- Exclude on topic
  *Exclude if the topic is about other stuff not relevant for the study*
- Exclude on type
  *Exclude if study is a policy analysis or opinion piece*
- Include based on full text
  *Cannot be excluded so is marked as included. Will require retrieval of full report.*
- General exclusion
  *Irrelevant studies*
- Only impact of CBHI

*Exclude studies which measure impact of CBHI schemes.*

1. Third screening (251)

- Included on mutual consent
  *Inclusion based on independent screening.*
- Excluded as private/SHI/Ghana NHIS, etc.
- Excluded as not determinants.
- Policy brief
- Excluded as could not be found
- Excluded as willingness to pay only

1. Data extraction tool (62 studies)

- Identification of report
  *How the report has been identified.*
  - Name of study
  - Author
    - Name
    - Affiliation
      *Academic organization or consultant*
    - Country
      *Country of author*
  - Which search strategy was used to identify this report?
    - Online databases
      *EconLit, PubMed, etc.*
    - Hand search
      *The report was found through hand searching a journal.*
    - Citation
      *The report was identified from the bibliographical list of another report.*
    - Contact
      *Through personal or professional contact.*
    - Unknown
      *Source Unknown*
  - Status of report
    - Published
      *If the report has an ISBN ISSN number.*
    - In press
      *Accepted for publication, but yet not published.*
    - Unpublished
      *If it does not have an ISSN ISBN number.*
  - Linked items
    *If this report is linked to one or more other reports in such a way that they also report the same study.*
    - Not linked
    - Linked
      *Details of bibliography or unique identifier.*
  - Language of report
    - English
    - Other (specify)
- Study details
  - Study type
    - Quantitative
    - Mixed methods
    - Undecided
    - Case study finding
    - Qualitative
  - Study design
    - Random controlled trials
    - Cohort studies
      - Yes
      - No
      - Unclear
    - Case control
      - Yes
      - No
      - Unclear
    - Cross-sectional studies
      - Yes
      - No
      - Unclear
    - Case SERIES AND CASE REPORTs.
      - Yes
      - No
      - Unclear
    - Ideas, opinions, editorials anecdotal.
      - Yes
      - No
      - Unclear
    - Descriptive
    - Review/systematic review
    - Quasi experimental
    - Theoretical study
    - Assumption
      - Theoretical assumptions
        *For qualitative studies only.*
    - Aim
      - Aim or objective
- Study Setting
  - Country

*Those Countries where the study was carried out. If the study was conducted in more than one country then all the countries will be included.*

- - - Region
    - Time period for which the study was conducted
    - Any
    - Specific
    - Not mentioned
  - Population studied
  - Scheme
    - Scheme details
- Intervention
  - Type of Intervention.
    - Voluntary.
    - Community participation.
      - Yes
      - No
  - Type of participants
    - - Members voluntarily chose to join the scheme.
      - Members voluntarily chose not to join the scheme.
      - Members chose to affiliate or re-affiliate.
  - Scale and size of scheme
    - - Local
      - Regional
      - National
      - International
  - Equity
    - - Poverty/Income
      - Geography
      - Gender
      - Age
      - Health status
- Methodology
  - Validity
    - Heterogeneity
      *Are the following sub-group effect considered?*
      - Yes
        - Age group
        - Women
        - Socioeconomic status
        - Geographically remote areas
      - No
      - Unclear
  - Analysis
    - Main analysis of the report
  - Data Collection
    - Data collection technique
  - Sample
    - Sampling and number of participants
- Domains evaluated
  - Enabling and limiting factors
    - Household characteristics
      - Household income
        - Positive
        - Negative
        - No effect
      - Household Size
        - Positive
        - Negative
        - No effect
      - Health expenses
        - Positive
        - Negative
        - No effect
      - Health events
        - Positive
        - Negative
        - No effect
      - Women below age 40
        - Positive
        - Negative
        - No effect
      - Number of children and aged
        - Positive
        - Negative
        - No effect
      - Education
        - Positive
        - Negative
        - No effect
      - Risk perspective
        - Positive
        - Negative
        - No effect
      - Understanding of benefits packages
        - Positive
        - Negative
        - No Effect
      - Female-headed household
        - Positive
        - Negative
        - No effect
      - Elderly headed
        - Positive
        - Negative
        - No effect
- Others
  - - Social capital
      - Trust in insurance scheme provider/management
        - Positive
        - Negative
        - No effect
      - Broad Image of the intermediary
        *NGO provider, MFI, etc.*
        - Positive
        - Negative
        - No effect
      - Risk sharing and solidarity
    - Scheme-related factors
      - Benefits package design
        - Positive
        - Negative
        - No effect
      - Premiums
        - Positive
        - Negative
        - No effect
      - Procedure for claim settlement
        - Positive
        - Negative
        - No effect
      - Good quality of service delivery
        - Positive
        - Negative
        - No effect
    - Institutional factors
      - Regulatory mechanism
        - Positive
        - Negative
        - No effect
      - Aspects relevant for setting up a local, self-run health insurance plan
        - Positive
        - Negative
        - No effect
      - Membership of self-help group
        - Positive
        - Negative
        - No effect
      - Marketing
        - Positive
        - Negative
        - No effect
      - Availability of subsidy
        - Positive
        - Negative
        - No effect
    - Supply-side factors
      - Availability of Healthcare
        - Positive
        - Negative
        - No effect
      - Quality of care
        - Positive
        - Negative
        - No effect
      - Distance to healthcare
        - Positive
        - Negative
        - No effect
      - Understanding of scheme by officials
        - Positive
        - Negative
        - No effect
- Qualitative reporting

*Reporting from the qualitative studies.*

- Conclusion from the study
  *Main study findings.*
  - Code for conclusion
